# Applications of Alginate-Based Nanomaterials in Enhancing the Therapeutic Effects of Bee Products

**DOI:** 10.3389/fmolb.2022.865833

**Published:** 2022-04-11

**Authors:** Mohammad A. I. Al-Hatamleh, Walhan Alshaer, Ma’mon M. Hatmal, Lidawani Lambuk, Naveed Ahmed, Mohd Zulkifli Mustafa, Siew Chun Low, Juhana Jaafar, Khalid Ferji, Jean-Luc Six, Vuk Uskoković, Rohimah Mohamud

**Affiliations:** ^1^ Department of Immunology, School of Medical Sciences, Universiti Sains Malaysia, Kota Bharu, Malaysia; ^2^ Cell Therapy Center (CTC), The University of Jordan, Amman, Jordan; ^3^ Department of Medical Laboratory Sciences, Faculty of Applied Medical Sciences, The Hashemite University, Zarqa, Jordan; ^4^ Department of Medical Microbiology and Parasitology, School of Medical Sciences, Universiti Sains Malaysia, Kota Bharu, Malaysia; ^5^ Department of Neurosciences, School of Medical Sciences, Universiti Sains Malaysia, Kota Bharu, Malaysia; ^6^ School of Chemical Engineering, Engineering Campus, Universiti Sains Malaysia, Nibong Tebal, Malaysia; ^7^ Advanced Membrane Technology Research Centre (AMTEC), School of Chemical and Energy Engineering, Faculty of Engineering, Universiti Teknologi Malaysia, Skudai, Malaysia; ^8^ LCPM, CNRS, Université de Lorraine, Nancy, France; ^9^ TardigradeNano LLC, Irvine, CA, United States

**Keywords:** alginates, alginic acid, honey, propolis, green synthesis, nanomedicine, nanobiotechnology, regenerative medicine

## Abstract

Since the ancient times, bee products (i.e., honey, propolis, pollen, bee venom, bee bread, and royal jelly) have been considered as natural remedies with therapeutic effects against a number of diseases. The therapeutic pleiotropy of bee products is due to their diverse composition and chemical properties, which is independent on the bee species. This has encouraged researchers to extensively study the therapeutic potentials of these products, especially honey. On the other hand, amid the unprecedented growth in nanotechnology research and applications, nanomaterials with various characteristics have been utilized to improve the therapeutic efficiency of these products. Towards keeping the bee products as natural and non-toxic therapeutics, the green synthesis of nanocarriers loaded with these products or their extracts has received a special attention. Alginate is a naturally produced biopolymer derived from brown algae, the desirable properties of which include biodegradability, biocompatibility, non-toxicity and non-immunogenicity. This review presents an overview of alginates, including their properties, nanoformulations, and pharmaceutical applications, placing a particular emphasis on their applications for the enhancement of the therapeutic effects of bee products. Despite the paucity of studies on fabrication of alginate-based nanomaterials loaded with bee products or their extracts, recent advances in the area of utilizing alginate-based nanomaterials and other types of materials to enhance the therapeutic potentials of bee products are summarized in this work. As the most widespread and well-studied bee products, honey and propolis have garnered a special interest; combining them with alginate-based nanomaterials has led to promising findings, especially for wound healing and skin tissue engineering. Furthermore, future directions are proposed and discussed to encourage researchers to develop alginate-based stingless bee product nanomedicines, and to help in selecting suitable methods for devising nanoformulations based on multi-criteria decision making models. Also, the commercialization prospects of nanocomposites based on alginates and bee products are discussed. In conclusion, preserving original characteristics of the bee products is a critical challenge in developing nano-carrier systems. Alginate-based nanomaterials are well suited for this task because they can be fabricated without the use of harsh conditions, such as shear force and freeze-drying, which are often used for other nano-carriers. Further, conjunction of alginates with natural polymers such as honey does not only combine the medicinal properties of alginates and honey, but it could also enhance the mechanical properties and cell adhesion capacity of alginates.

## Introduction

Already in the ancient times, when there was a shortage or complete absence of pharmacotherapies, bee products emerged as trusted natural medicines for ameliorating a wide range of illnesses (Kuropatnicki et al., 2013; Pan et al., 2014). The diverse range of bee products includes honey, propolis, pollen, bee venom, bee bread, and royal jelly (Cornara et al., 2017). Although the source of all of these products is bee, they differ in their composition. The composition of different bee products varies depending on bee species, the surrounding vegetation, geographic location, and seasonality (Kocot et al., 2018). Hence, the medicinal properties of these products can be varied too. Scientists have conducted a lot of research to explore the therapeutic properties of each of these products as produced by different bee species and in different regions (Rao et al., 2016; Kocot et al., 2018; Al-Hatamleh et al., 2020a; Nainu et al., 2021).

Over the past few decades, a number of research studies have evaluated the bioactivities and therapeutic properties of bee products, showing promising findings. These findings revealed a plenty of antioxidant, anti-inflammatory, antimicrobial and anticancer effects of bee products (Al-Hatamleh et al., 2020a; Al-Hatamleh et al., 2020b; Nainu et al., 2021). Further, several reports indicated that bee products have promising effects such as antidiabetic, wound healing, anti-hepatotoxicity, anti-aging, anti-ulcer, and cardioprotective among others (Ahmed et al., 2017; Fazalda et al., 2018; Nainu et al., 2021). The rich chemical composition of bee products includes essential nutrients, sugars, minerals, and vitamins, which all contribute to endowing bee products with beneficial effects (Ajibola et al., 2012; Pasupuleti et al., 2017; Durazzo et al., 2021). However, studies have indicated that this wide range of biological activities is mainly attributable to the high antioxidant (phenolic compounds and flavonoids) content of bee products (Al-Hatamleh et al., 2020a; Durazzo et al., 2021).

**FIGURE 1:**
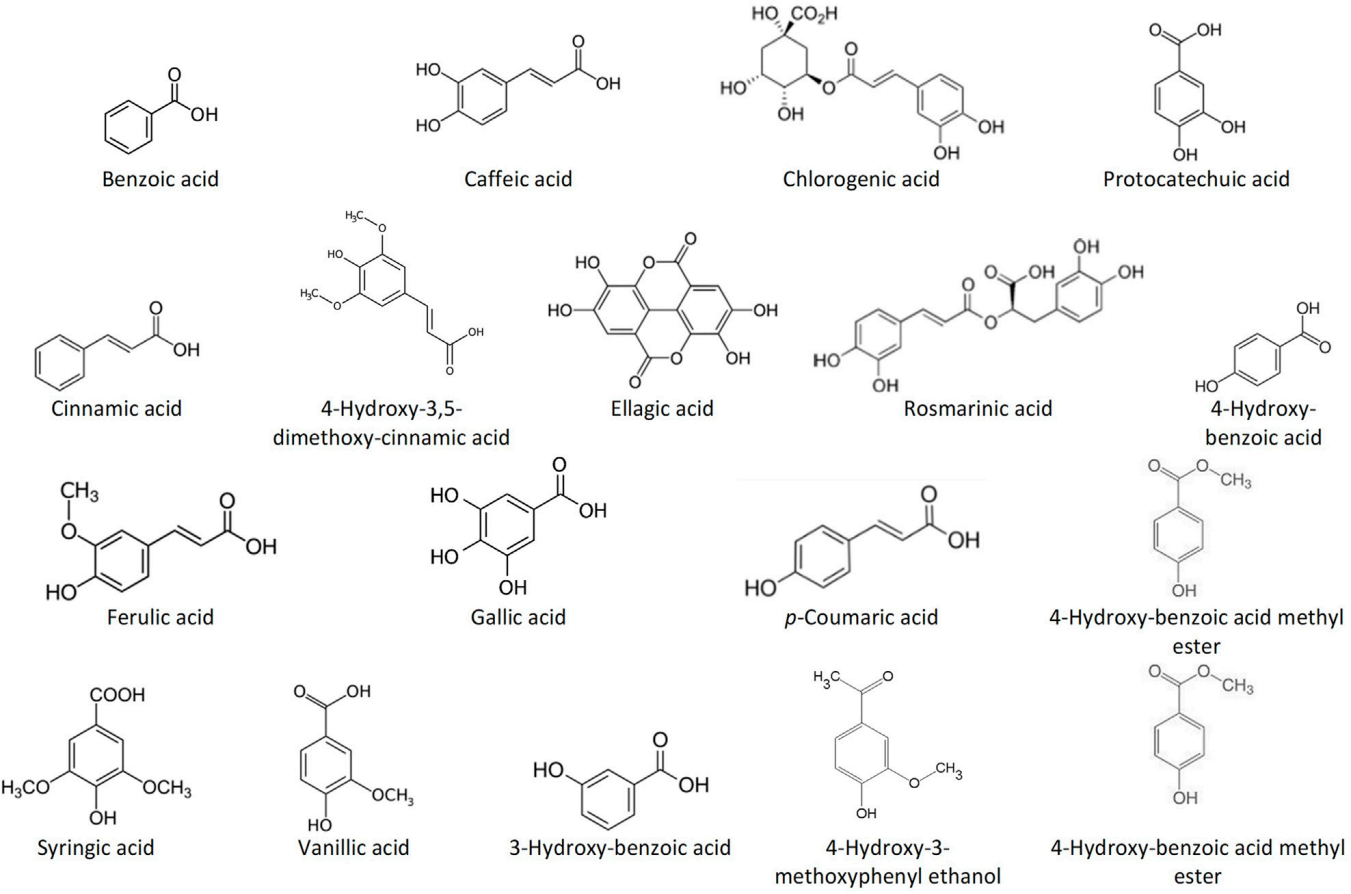
Structures of the most common phenolic acids in bee products.

**FIGURE 2:**
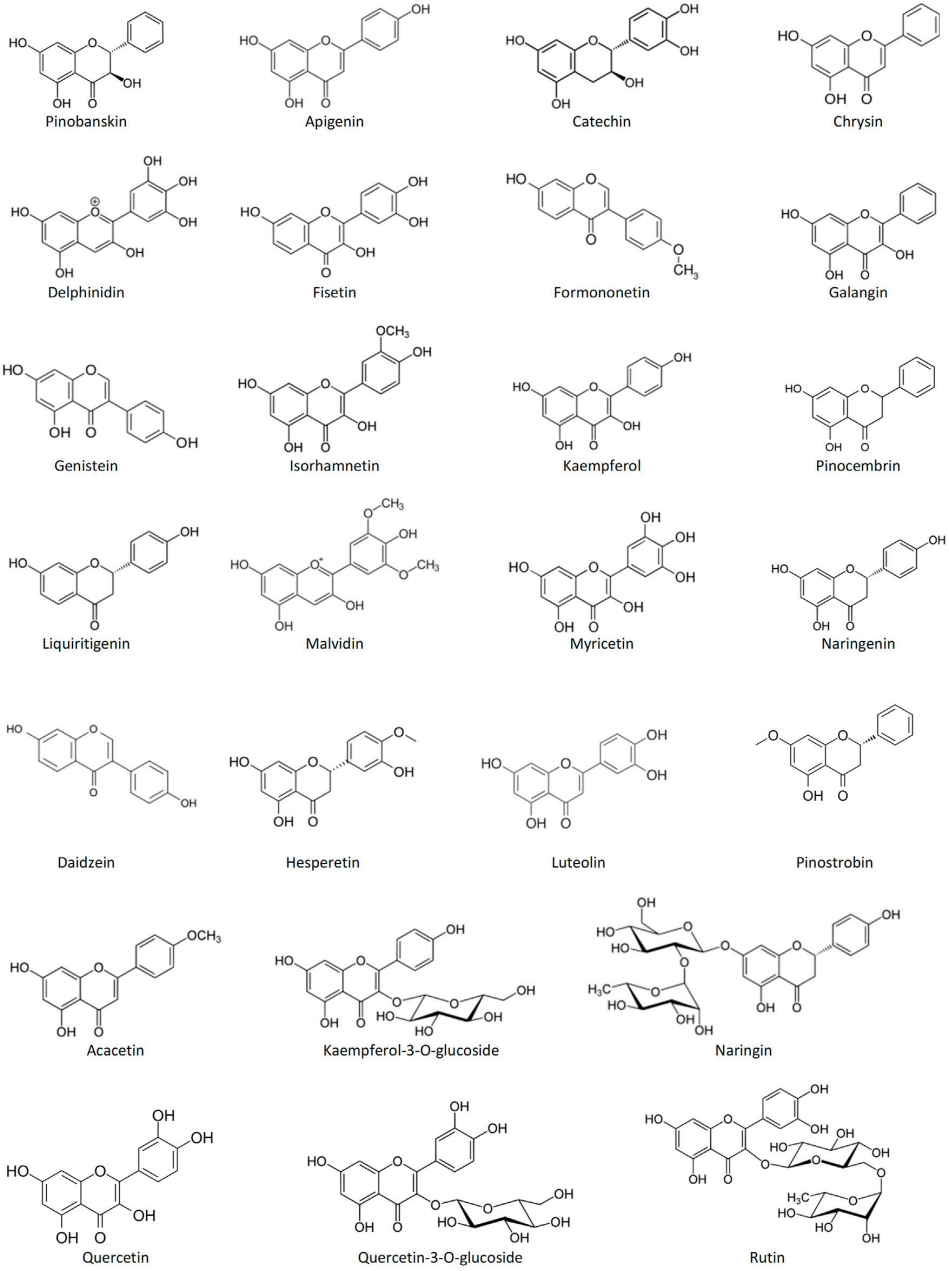
Structures of the most common flavonoids in bee products.

Among the most common phenolic acids in bee products are benzoic acid, caffeic acid, chlorogenic acid, cinnamic acid, ellagic acid, ferulic acid, gallic acid, *p*-coumaric acid, protocatechuic acid, rosmarinic acid, syringic acid, vanillic acid, 3-hydroxy-benzoic acid, 4-hydroxy-benzoic acid, 4-hydroxy-benzoic acid methyl ester, 4-hydroxy-3-methoxyphenyl ethanol, and 4-hydroxy-3,5-dimethoxy-cinnamic acid (Stalikas, 2007; Alvarez-Suarez, 2017; Kocot et al., 2018) (Figure 1). Bee products are also rich in flavonoids such as acacetin, apigenin, catechin, chrysin, daidzein, delphinidin, fisetin, formononetin, galangin, genistein, hesperetin, isorhamnetin, kaempferol, kaempferol-3-O-glucoside, luteolin, liquiritigenin, malvidin, myricetin, naringenin, naringin, pinobanskin, pinocembrin, pinostrobin, quercetin, quercetin-3-O-glucoside, and rutin (Stalikas, 2007; Alvarez-Suarez, 2017; Kocot et al., 2018) (Figure 2). The antioxidant molecules (phytochemicals) present in bee products can assist in coping with oxidative stress, which is implicated in most human diseases (Martinello and Mutinelli, 2021). Oxidative stress is the state marked by an elevated production of free radicals, reactive oxygen species (ROS) and reactive nitrogen species (RNS), resulting in an imbalance between free radicals and antioxidants, and subsequently impaired physiological functions (Al-Hatamleh et al., 2017; Pizzino et al., 2017; Al-Hatamleh M. A.İ. et al., 2020).

Hence, phytochemicals in bee products have expressed vital modulatory effects on many essential molecules involved in the pathogenesis of various human diseases, such as nuclear factor kappa B (NF-κB), glutathione, activator protein 1 (AP-1), protein kinase C (PKC), and cyclooxygenase-2 (COX-2) (Park et al., 2010; Seo et al., 2018; Talebi et al., 2020; Mohany et al., 2022). However, most studies have used *in-vitro* models to examine the bioaccessibility and bioavailability of phytochemicals from bee products (Yesiltas et al., 2014; Aylanc et al., 2021b). In various animal and clinical trials, the administration of bee products was limited to using them as dietary supplements or topical treatments (Bergman et al., 1983; Samarghandian et al., 2017; Luo et al., 2021). Thus, due to differences in conditions and concentrations, the observed effects may not be the same as those produced by *in-vivo* clinical trials. For example, phytochemical-derived metabolites are affected by the gastric and hepatic processes and their bioavailability varies depending on their source (Ranneh et al., 2021). Although no side effects have been reported for phytochemicals from bee products, the bioavailability and pharmacokinetic profiles of these molecules vary due to differences in absorption and systemic elimination when they are used in their crude forms (Koh et al., 2020; Aylanc et al., 2021a; Becerril-Sánchez et al., 2021). Hence, these factors may hamper the therapeutic applications of bee products.

On the other hand, the growing demand for using natural products in the pharmaceutical and food industries has led researchers to focus on the development of green synthesized nanomaterials (Gour and Jain, 2019; Jadhav and Kokate, 2021). Recent years have witnessed the establishment of a variety of applications and approaches that utilize nanomaterials to enhance the therapeutic efficacy and bioavailability of bee products (Tatli Seven et al., 2018; Bonsignore et al., 2021). Also, bee products have often relied on nano-formulations, not only to reduce the potential toxicity of concurrently loaded drugs but also to enhance the bioaccessibility and bioavailability (Balasooriya et al., 2017; Khongkaew and Chaemsawang, 2021). To study the effectiveness of bee products in targeting the physiological sites of different diseases (especially tumors and inflammations), different types of nanomaterials, such as lipids and polymers, and the self-nano-emulsifying drug delivery systems were used in loading and delivery of bee products or their extracts (Fan et al., 2013; Ilhan-Ayisigi et al., 2020; Tatli Seven et al., 2020; Ullah et al., 2020; Fitria et al., 2021). The bee products-incorporated nano-objects have shown higher bioactivity, bioaccessibility, and physical and chemical stability than the crude products. Further, nanotechnology applications have allowed the loading of specific compounds extracted from the bee products with more predictable therapeutic effects for use in targeted therapies (Patra et al., 2018). Towards keeping the bee products as natural and non-toxic therapeutics, the green synthesis of nanocarriers loaded with these products or their extracts has received a special attention (Sharma et al., 2019; Bonsignore et al., 2021; Shreyash et al., 2021). Owing to their biocompatibility, biodegradability, stability, sustainability, and ability to control the release of loaded therapeutics, naturally derived polysaccharides have emerged as efficient polymeric nano-carriers (Nitta and Numata, 2013; Maiti and Jana, 2019; Jana et al., 2021). Furthermore, polysaccharides have allowed for flexibility in establishing chemical modifications designed to modulate specific size and surface properties of the products (Hasnain et al., 2020b). Natural polysaccharide nanomaterials have proven to be well-suited for protecting small and large molecules as well as biologics against the cellular degradation and environmental hazards (Hasnain et al., 2020b). Their reactive functional groups represent a cell-adhesive polymer surface on which cells can interact and adhere through passive and active adhesion, enabling improved drug retention time, absorption, and intracellular bioavailability (Hasnain et al., 2020b).

Alginate has been one of the most studied polysaccharides. Alginates are naturally occurring hydrophilic polysaccharides extracted from the seaweeds or obtained *via* bacterial biosynthesis (Hasnain et al., 2020a). Over the past few decades, several forms of alginate-based systems have been fabricated and examined for use in a variety of different pharmaceutical and biomedical applications such as controlled drug delivery, wound dressings, tissue engineering, tissue regeneration, and dental impressions (Hasnain et al., 2020a). Alginate-based nanomaterials are among the most extensively characterized biopolymers used to develop targeted or localized delivery systems for therapeutic compounds, including bee products or their extracts (Abasalizadeh et al., 2020; Homem et al., 2021; Saberian et al., 2021). This is due to their offering desirable characteristics that include a high therapeutic-payload, targeted efficiency, pH sensitivity, capability of protection from degradation, thickening properties, gelling abilities, high availability, and relatively low cost (Lee and Mooney, 2012; Sun and Tan, 2013; He et al., 2020).

**FIGURE 3:**
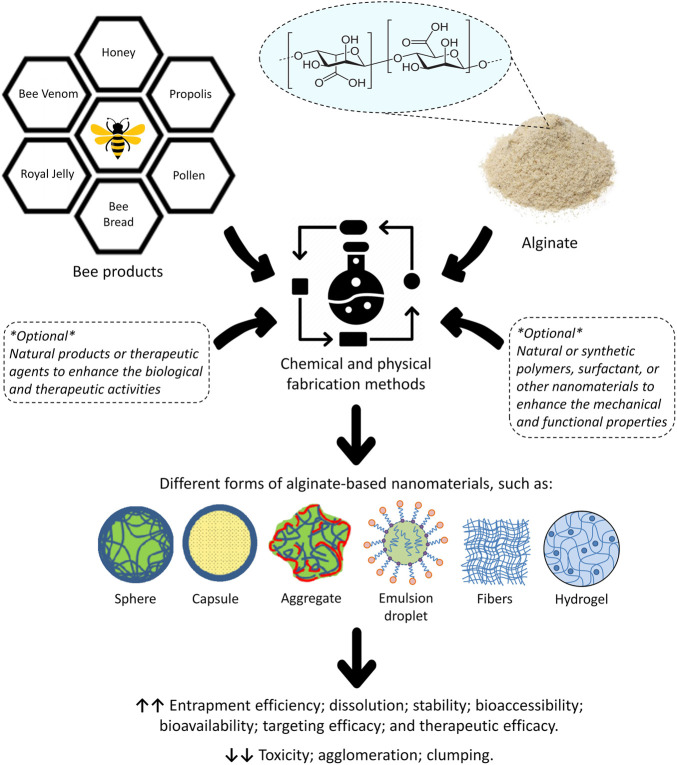
General overview of the potential role of alginate-based nanomaterials in enhancing the biological and therapeutic properties of bee products or their extracts, as well as reducing the toxicity, agglomeration and phase separation of the given nanomaterials.

Alginate-based nanomaterials for enhancing the therapeutic capabilities of bee products and keeping their physicochemical properties deserve a considerable attention as a promising pathway for industrial and therapeutic applications. Thus, the focus of this work is on providing an explicit review of various nanotechnology applications that utilize alginate-based nanomaterials to incorporate bee products or their extracts and thus enhance their therapeutic effectiveness (Figure 3). Literature search was conducted using different databases, including ScienceDirect, Web of Science, Scopus, PubMed, and Google Scholar, so as to identify all the relevant previous studies in the field. A wide range of keywords was used, including but not limited to alginate, alginic acid, natural products, biopolymers, marine polymers, nano-polysaccharides, nanobiotechnology, nanomaterials, bee, honeybee, stingless bee, honey, propolis, pollen, bee venom, bee bread, royal jelly, and regenerative medicine. To the best of our knowledge, this is the first review addressing this crucial topic, and therefore it will help researchers to further understand and develop novel alginate-based nanoformulations for loading bee products or their extracts, which eventually could be used in the commercial development of therapeutic, cosmetic, and nutritional products. Also, with this review, the readers can have a broad overview of this interesting research area spanning from the fundamentals to the potentials and advantages of alginate-based nanomaterials loaded with bee products.

## Chemistry and Physicochemical Properties of Alginate

**FIGURE 4:**
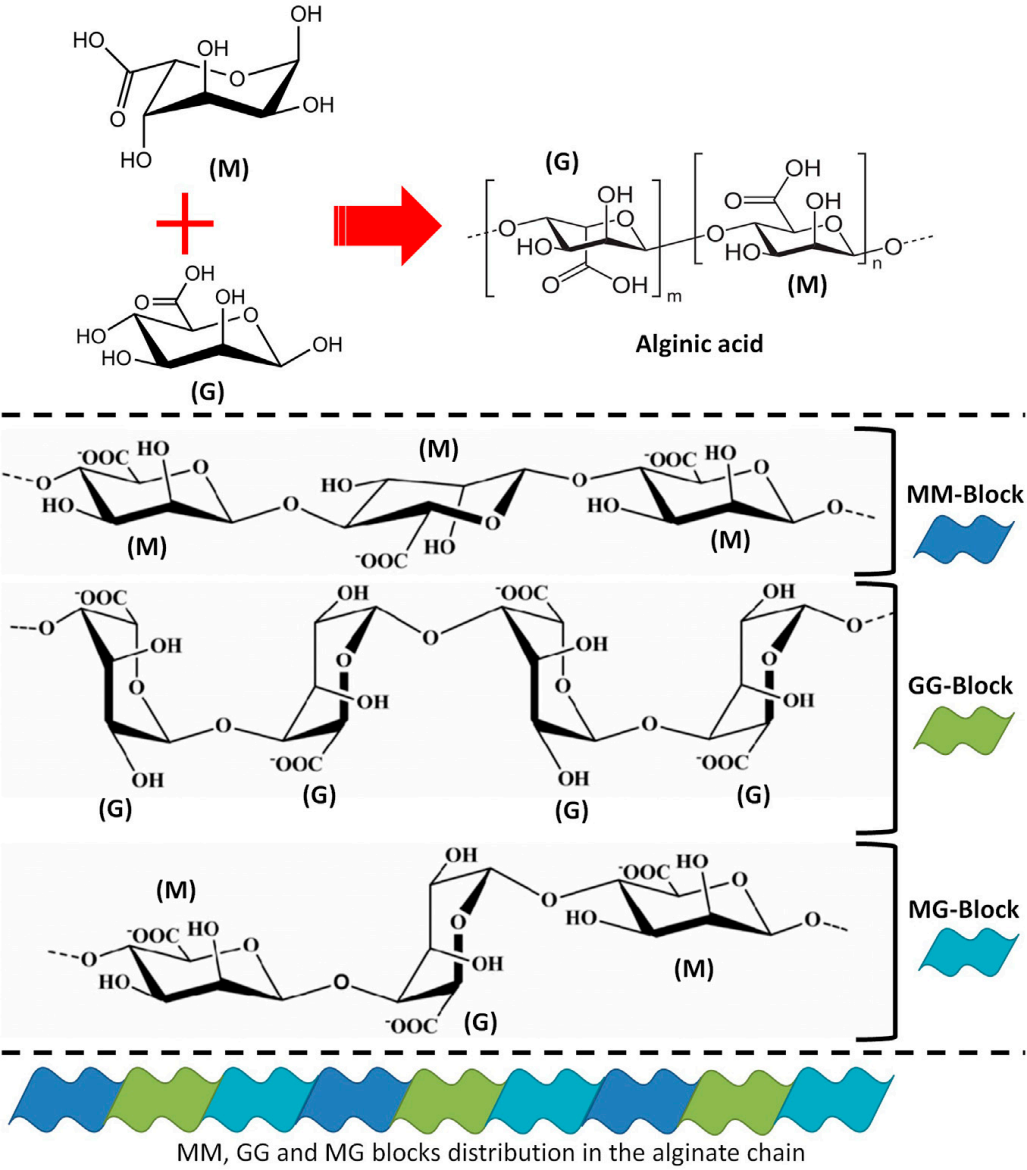
The structural details of alginate. (M) refers to D-mannuronic acid and (G) refers to L-guluronic acid.

Alginate, also known as alginic acid or algin, is a naturally occurring anionic polysaccharide extracted from brown algae (seaweeds or *Phaeophyceae*), including *Macrocystis pyrifera*, *Laminaria hyperborea*, *Ascophyllum nodosum*, *Laminaria japonica*, and *Laminaria digitata* (Hariyadi and Islam, 2020). Alginate was first described, extracted, and patented in the 1880s by the British chemist E. C. C. Stanford, who named it “algin” (Hasnain et al., 2020a). The molecular structure of alginate consists of linear blocks co-polymers with β-(1→4)-linked D-mannuronic acid (M) and α-(1→4)-linked L-guluronic acid (G) residues (Rosiak et al., 2021). There are three forms of segments of the polymer blocks, varying based on the type of the brown algae that alginate was extracted from. These different forms differ in the proportion of M and G residues; homopolymeric blocks of M residues (MM blocks) or G residues (GG blocks); and heteropolymeric sequences of randomly coupled both M and G residues (MG or GM blocks) (Zhang H. et al., 2021) (Figure 4). Alginate is also presents as capsular polysaccharides in some bacteria such as *Burkholderia pseudomallei*. Also, a high osmolarity can trigger *Pseudomonas aeruginosa* to produce alginate as a consequence of desiccation (Draget and Taylor, 2011; Reckseidler-Zenteno, 2012). The polymer segments of alginate extracted from bacteria consist of up to 100% M residues (homopolymeric blocks of M) (Ertesvåg et al., 1996). However, alginate extracted from microbial sources is still not industrially available, whereas algae remain typically the main commercial source (Draget and Taylor, 2011).

The commercially available alginate is typically extracted from the brown algae cell walls as water-insoluble alginic acid, after which these salts are converted to soluble and purified sodium alginates (Łabowska et al., 2019). Chemical pretreatment with 0.1% formaldehyde is the first step and a necessary one to eliminate any pigments. To provide optimal acid pretreatment conditions, diluted hydrochloric acid (HCl) is used to dissolve alginate, removing its counter ions and any soluble impurities, thus increasing the yield of alginate (Hernández-carmona et al., 1998). Secondly, an alkaline solution of sodium carbonate (Na_2_CO_3_) or sodium hydroxide (NaOH) is added to the harvested insoluble alginate and then filtered to accelerate the formation of alginate in an aqueous solution (Zhang H. et al., 2021). Lastly, precipitation of alginate from the filtered solution, either as alginic acid, sodium alginate, or calcium alginate is accomplished by adding dilute HCl, ethanol, or calcium chloride (CaCl_2_), respectively (Arvizu-Higuera et al., 1997; Zhang H. et al., 2021). Further purification and conversion processes as needed are applied on the precipitates, after which it is separated, dried, and milled.

**FIGURE 5:**
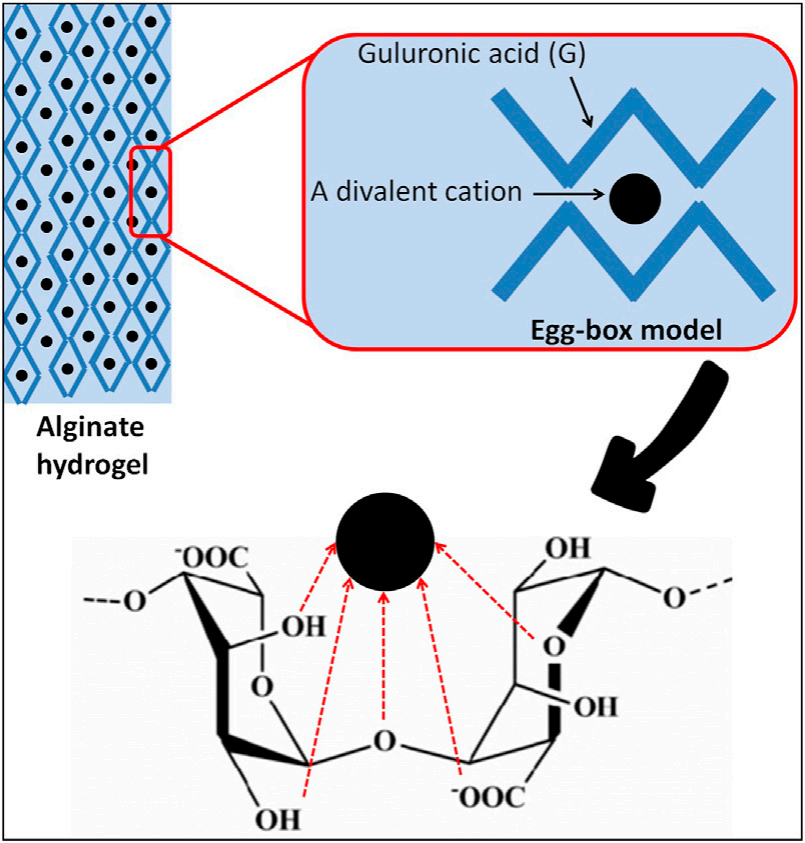
Egg-box model-based gelation of alginates.

Alginates possess unique nontoxic and functional properties, including water absorption and retention, thickening and gelling, but also the ability to stabilize and increase the viscosity of liquids (Lee and Mooney, 2012; Cervino et al., 2018). Alginates are suitable polymers for forming hydrogels. The gelling mechanism involves complexation of divalent cations (e.g., Ca^2+^, K^+^, Na^+^, and Mg^2+^) with blocks of G residues in the polymer chain by cross-linking its carboxyl groups sequences (Abasalizadeh et al., 2020), which is conventionally described by the “egg-box” model (Grant et al., 1973) (Figure 5). Studies have shown that the amount and distribution of the G residues are the most important properties that influence the gelling process of alginates (Hongu et al., 2005; Jeong et al., 2020). Gelation of alginates requires a sufficient G residue content of a minimum 20–25% (Khotimchenko et al., 2001). While the ratio of M to G residues is positively correlated with the production of more elastic alginate gels, the reverse of this ratio correlates with the production of stiffer gels (Pragya et al., 2021).

Moreover, depending on the pH of the aqueous solution, the presence of a co-solvent (e.g., ethanol), the presence of divalent ions that enhance the gelation process, the ionic strength of the dissolving medium, the presence of gelation-promoting ions, and the structural and mechanical properties of the biopolymer, alginate gels can have different viscosities, strengths, and solubility and they can be even water-insoluble (Gurikov and Smirnova, 2018; Rosiak et al., 2021). Unlike other polysaccharides sourced from algae, such as agar and carrageenans, alginate gels are thermally irreversible; they cannot be heat-solubilized and are repeatedly formed by cooling (2014; Rhein-Knudsen et al., 2015). Based on the “egg-box” model, it became possible to understand the capability of alginates to selectively bind metallic cations. Due to its high molecular weight (100–500 kDa), sodium alginate is water-soluble, but calcium alginate is insoluble and swells in water, whereas alginic acid is insoluble in water, and yet it binds to it (Saitoh et al., 2000; Kim et al., 2007; Rosiak et al., 2021). Studies have shown that alginate-based films are uniform, transparent, impermeable to oils and fats, water-soluble, good oxygen barriers, and have a high water vapor permeability (WVP) (Rhim, 2004; Singh et al., 2020; Kocira et al., 2021). It has been reported that the WVP is significantly affected by the alginate composition and films with a higher proportion of G residues proved to be better moisture barriers (Olivas and Barbosa-Cánovas, 2008). In contrast, the poor water resistance of alginate films is attributed to their hydrophilic nature (Singh et al., 2020). In addition, alginate films possess other desired properties including their antioxidant, antibacterial, and anti-lipid peroxidation capacities, as well as preserving original characteristics such as flavor and color (Alves et al., 2021). Thus, due to all these biological properties alginates have been selected as ideal vehicles for carrying molecules and drugs in pharmaceutical applications as well as for food packaging applications.

## Alginate Nanoformulations

Production of alginate-based nano-carrier systems has been successfully adopted based on various preparation methods. Choosing an appropriate alginate-based nanoformulation depends upon various factors. Basically, the nature of loaded molecules or therapeutic agents and the goal of the nano-delivery system, which in turn determine the required characteristics of the nanomaterial and the route of administration, can guide for identifying the suitable alginate-based nanoformulation (Choukaife et al., 2020). Most of the alginate-based nanomaterials developed for pharmaceutical and biomedical applications are in the form of hydrogels, which are networks of three-dimensional (3D) cross-linked water-swollen polymers (Lee and Mooney, 2012). Nano-hydrogels of alginates can be fabricated through several strategies, including ionic crosslinking, covalent crosslinking, thermoresponsive phase transition (thermal gelation), cell crosslinking, free radical polymerization, and “click” chemistry (Abasalizadeh et al., 2020).

Due to the simple procedure, involving no potentially toxic catalysts, but also overall biocompatibility, reversibility, and the ability to provide rapid dissolution for recovery and analysis, ionic crosslinking has been the most common method to produce alginate hydrogels. This method requires divalent cations to cooperatively interact with G-monomer building blocks to form ionic bridges during alginate gelation (Agulhon et al., 2012). The following divalent cations are commonly used to produce ionically cross-linked alginate hydrogels: Ca^2+^, Mg^2+^, Fe^2+^, Ba^2+^, and Sr^2+^. Here, CaCl_2_ is known as one of the best and most frequently used alginate crosslinking agents (Zhang C. et al., 2021; Zhang H. et al., 2021). However, the major disadvantages of ionic crosslinking are pH-responsive swelling properties, poor mechanical properties, and the risk of dissolution of the hydrogel system. In general, alginate hydrogels exhibit high biocompatibility and oxygen permeability. Encapsulation of cells into alginate hydrogels, which resemble natural soft tissue, facilitates the transport of nutrients into and cellular waste out of the hydrogels. This makes them attractive scaffolds for tissue engineering and other pharmaceutical and biomedical applications (Zhu and Marchant, 2011; Aswathy et al., 2020). Furthermore, due to the presence of many carboxyl groups located on the polymer backbones, alginate hydrogels exhibit pH-responsive swelling/contraction properties (Sun and Tan, 2013).

To produce alginate-based nanoparticles (NPs) loaded with various compounds, several fabrication techniques have been developed including emulsification-gelation, emulsification-solvent displacement, emulsion-solvent evaporation, complexation, layer-by-layer approach, spray drying, and electrospraying (electrohydrodynamics atomization) (Choukaife et al., 2020). The emulsification-gelation technique depends on the encapsulation of oil droplets within the hydrogels, forming alginate-based oil-in-water (O/W) emulsion systems, during crosslinking through external or internal gelation (Ong et al., 2015; Zhang C. et al., 2021). This technique can be cost-effective because high payloads in the oil phase could be achieved with a lesser use of the encapsulated material (Chan, 2011). In the external gelation technique, the divalent cation, for example Ca^2+^, is insoluble in the oil phase and it diffuses from the external continuous phase into the inner structure of alginate emulsion droplets to react immediately with the carboxylic groups of G residues (Auriemma et al., 2020). In the internal gelation, the Ca^2+^ release through pH reduction initiates gelation of the dispersed alginate droplets due to migration of Ca^2+^ ions from the inner droplets to the outer part (Ahmed et al., 2013; Yom-Tov et al., 2015). Regarding the emulsification-solvent displacement as a form of nanoprecipitation, this technique is based on the rapid diffusion of the solvent from the internal phase to the external phase, which thereby provokes the aggregation of the polymer and the loaded compound in the form of colloidal NPs (Esmaeili et al., 2007; Choukaife et al., 2020). Compared to the general O/W emulsion encapsulation, the organic solvent is removed by evaporation during the emulsification-solvent evaporation process, while the drug and the polymer are precipitated in the droplets, thus forming the nanocapsules or nanosphere (Hoa et al., 2012). Since this requires relatively mild operating conditions (e.g., continuous stirring at ambient temperature), this technique is preferred over other fabrication methods, such as homogenization, spray drying, and sonication; in order to produce a stable emulsion without compromising the activity of the loaded compound or drug (Hoa et al., 2012).

Spray drying is an extensively used technique in biopolymer encapsulation of bioactive ingredients. This method has a great potential as it allows the integrity of compounds to be preserved during subsequent processing and digestion (Bagheri et al., 2014). This technique was successfully used to encapsulate peptides and proteins into alginate micro- and nano-particles with a high encapsulation efficiency and good functional properties (De Cicco et al., 2014; Dhamecha et al., 2019). Briefly, a hot gas stream leads to evaporation of atomized droplets of a continuous polymer/protein feed, encapsulating the drug. The surface temperature of the droplet reaches the temperature of the drying air and the drying rate increases with an increase in drying temperature. Due to continuous evaporation of the water, the surface temperature of the droplet remains constant. Then, as a result of decreasing the drying rate, a crust forms encapsulating the molecules (Erdinc and Neufeld, 2011; Shehata and Ibrahima, 2019). Atomizing alginate solution by applying electrostatic forces stronger than its surface tension is also possible and it constitutes the electrospraying technique, which is also used to fabricate alginate-based NPs (Alallam et al., 2020). Due to the external force composed of attractive and repulsive electrostatic terms, a solution drop ejected at the capillary nozzle tip upon exposure to a high electric field undergoes a shape deformation to a cone. As a result of varicose instability, a fine jet that subsequently breaks up into drops is emitted from the cone’s tip (Nikoo et al., 2018). Electrospraying has advantages such as producing monodisperse alginate particles with a consistent and controlled size, shape, and encapsulation efficiency, while the incorporated biomolecules are not affected by the free charges that gather on the droplet surface (Faramarzi et al., 2017; Shaiqah et al., 2020). In addition, the technique is marked by ease of operation under optimum parameters and cost-effectiveness. Another technique used to fabricate alginate NPs is complexation, which depends on electrostatic interactions between alginate at neutral and alkaline pH values, but also with bioactive agents or compounds, surfactants, and other kinds of naturally occurring polymers that can promote the encapsulation efficiency and reduce the porosity, such as the polycation chitosan (Cardoso et al., 2016; Hariyadi and Islam, 2020). Furthermore, alginate bilayer coating is used in the fabrication of different kinds of NPs in order to prevent oxidation and aggregation, as well as to enhance their oral bioavailability through protection from intestinal degradation (Tsai and Ting, 2019; Kloster et al., 2021).

Moreover, the electrospinning technique allows for direct fabrication of alginate-based nanofibrous materials (Mokhena et al., 2020). Briefly, in this process, which is also known as a spinning technique, the electrostatic force is applied to the alginate liquid solution, which lets the solution extrude from a nozzle forming a jet. This jet is stretched and elongated to form alginate nanofibers during drying and deposited on the collector (Xue et al., 2019; Partheniadis et al., 2020). Mostly, alginates need to be fabricated with carrier polymers upon electrospinning, and only limited studies have examined the electrospinnability of alginate solution without employing carrier polymers (Bonino et al., 2011; Saquing et al., 2013). This is attributed to the high electrical conductivity of pure sodium alginate in an aqueous solution, which can be reduced by blending it with other polymers (Saquing et al., 2013). Alginate composite nanofibers were successfully produced by involving hydrosoluble polymers (e.g., polyvinyl alcohol) which enhances alginate electrospinnability by reducing viscosity, surface tension, and conductivity (Li et al., 2013). Other less commonly used routes for fabricating alginate nanofibers by electrospinning include incorporation of co-solvent systems (e.g., glycerol and water), which also improve alginate electrospinnability (Nie et al., 2008), as well as using carrier polymers with co-solvents and surfactants (e.g., DMSO and Triton, respectively) (Saquing et al., 2013) in order to increase the content of alginate and to promote the formation of a 3D network of nanofibers, which may result from the reduction of the surface tension present at high alginate content (Bonino et al., 2011; Velasco-Barraza et al., 2018). Owing to their highly porous structure and large specific surface area, alginate nanofibers mimic the extracellular matrix and enhance the proliferation of epithelial cells and the formation of new tissues (Sun and Tan, 2013; Morelli et al., 2021). Also, their nano-size and nanofibrous mesh structure enhance hemostasis of injured tissues and stimulate rapid fluid absorption. By maintaining a moist microenvironment and effectively preventing bacterial permeation, their properties such as the ability to enhance dermal drug delivery, cell respiration, and high gas penetration, ensure that alginate nanofibrous mats are able to speed up wound healing (Wang et al., 2019; de Oliveira et al., 2021).

## Alginate-Based Nanomaterials for Biomedical and Pharmaceutical Applications

**FIGURE 6:**
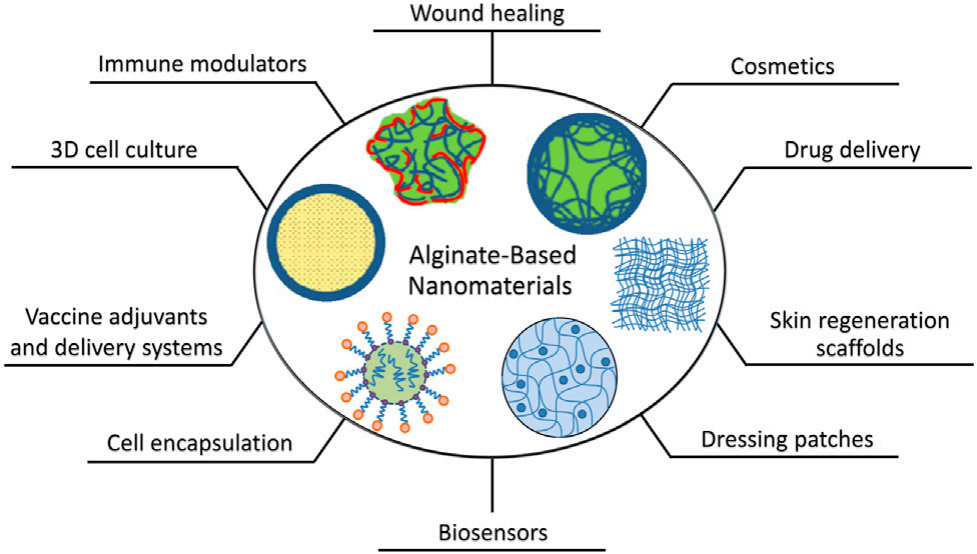
Schematics of the present and potential biomedical and pharmaceutical applications of alginate-based nanomaterials.

Over the last three decades, tremendous steps have been made towards producing alginate-based nanomaterials as biocompatible systems for advanced biomedical devices. These advancements have largely been made owing to the unique characteristics and pharmaceutical properties of alginate (Song et al., 2018; Anand and Rajinikanth, 2021) (Figure 6).

Owing to their ability to mimic the native extracellular matrix that supports cells by providing structural and biochemical conditions for cell attachment, proliferation, migration, and differentiation, alginate hydrogels are extensively involved in wound healing and tissue engineering when applied as scaffolds (Sun and Tan, 2013; Kalva et al., 2021; Nosrati et al., 2021). Alginate hydrogels have encouraged the wound healing process under different wound conditions and have been used to produce sponge-like products as wound dressing matrices (Kurakula et al., 2020). Currently, there are several types of alginate-based dressings that are commercially available for various types of wounds, for example Algicell^™^, AlgiSite M™, Comfeel Plus™, Kaltostat™, Tromboguard®, Algivon®, and Hyalogran® (Aderibigbe and Buyana, 2018). The mesh-like structure produced by the alginate hydrogels covers the wound surface and protects it from the microbial entry and, thereafter, from infection (Kurakula et al., 2020). Also, alginates are able to modulate the innate immune defense mechanisms. Through the NF-κB pathway, the stimulatory effect of alginate seems to involve an activation of macrophages present in large numbers in wound granulation tissue, playing a vital role in accelerating the wound healing process by promoting and resolving inflammation, removing dead cells and enhancing cell proliferation and tissue restoration (Yang and Jones, 2009; Krzyszczyk et al., 2018). Alginate has a stimulatory effect on monocytes, increasing the production of inflammatory cytokines, such as tumor necrosis factor (TNF), interleukin 6 (IL-6), and IL-1, which are necessary for intracellular signaling and thus for orchestrating the wound-healing process in some situations (Kulseng et al., 1996; Thomas et al., 2000; Eming et al., 2007).

Aside from tissue regeneration treatments, alginate biopolymers have been used in nanocomposite systems developed to overcome the issues arising from the obstructed oral delivery of drug-loaded NPs, including poor stability in different regions of the gastrointestinal tract and restriction by various biological barriers (Andretto et al., 2021). Such hybrid nanosystems could allow for the fabrication of novel systems with unique properties based on structural and mechanical modifications of both NPs and alginate polymeric matrices (Andretto et al., 2021). For example, alginate-encapsulated 2′-O-methyl-phosphorothioate antisense oligoribonucleotides adsorbed onto cationic core-shell NPs (ZM2-AON) proved effective upon the oral route administration in a mouse model of Duchenne’s muscular dystrophy (DMD) (Falzarano et al., 2013). The obtained nanocomposite system was proposed as an alternative to intraperitoneal injection of alginate-free ZM2-AON, which provoked dystrophin restoration in the muscles of mice (Bassi et al., 2012). The results reveled that alginate-encapsulated ZM2-AON induced only a slight dystrophin rescue in diaphragm and intestine smooth muscles, meaning that it could be a promising approach for the DMD treatment after further improvements (Falzarano et al., 2013). Moreover, alginate can also play a vital role in cytotherapy by incorporating different types of cells in alginate-based capsules (de Vos et al., 2014; Hariyadi and Islam, 2020). However, this approach seems to be still at the microcapsules level, not nanocapsules, and it could lead to promising directions in the future. Ciriza et al. have encapsulated genetically modified mesenchymal stem cells that secrete erythropoietin in hybrid alginate-protein-coated graphene oxide (GO) microcapsules and they confirmed that this improvement enhanced the cell survival and sustained the therapeutic protein release (Ciriza et al., 2018).

Due to their unique structures and biological properties, alginate-based nanocarriers have been utilized in pharmaceutical research for developing drug-delivery and coating systems. The investigated systems were designed for either controlled or sustained release delivery and through various administration routes, including transdermal, oral, nasal, mucosal, ocular, parenteral, and pulmonary (Niculescu and Grumezescu, 2022). Interestingly, all these systems have improved the drug entrapment efficiency, dissolution, bioaccessibility, bioavailability, and stabilization against degradation (Hariyadi and Islam, 2020). Alginate-based nanocarriers are well-known as potential oral delivery systems for insulin. In addition to the general advantages of alginate as a natural polymer, studies have suggested high association of insulin with it and have also indicated high encapsulation efficiency and optimum blood compatibility of alginate-based nanomaterials, as well as the capacity to preserve the insulin stability and bioavailability (Goswami et al., 2014; Patil and Devarajan, 2016). In cancer research, alginate-based nanomaterials emerged based on their potential to reduce the side effects of anti-cancer chemotherapeutic agents and enhance their efficacy in targeting and penetrating the target cells. They can also be used to overcome the chemotherapeutic resistance, which could result from several intracellular pathways of drug uptake and a higher accumulation of drug inside cancer cells (Alfarouk et al., 2015; He et al., 2020). For instance, Katuwavila et al. have encapsulated doxorubicin (DOX) in chitosan-alginate NPs, which were cross-linked through the ionic gelation method. The *in-vitro* assays on MCF-7 breast cancer cells indicated that the involvement of alginate resulted in dose and time-dependent cytotoxic activity, as well as a superior sustained release of DOX (Katuwavila et al., 2016).

Alginate-based nanomaterials are also considered as promising adjuvant and vaccine delivery systems (Sarei et al., 2013; Mosafer et al., 2019). For example, Sarei et al. used alginate NPs (70 ± 0.5 nm) for diphtheria toxoid (DT) delivery in pigs (Sarei et al., 2013). DT is a formaldehyde-inactivated purified preparation of inactivated diphtheria toxin (Metz et al., 2020). The DT-loaded alginate NPs showed high loading capacities (>90%) and a prolonged release profile. The DT-loaded alginate NPs were prepared by ionic gelation method, which did not affect the antigenic integrity and activity of DT, and they showed a higher humoral immune response than conventional vaccine (Sarei et al., 2013). In another study, Mosafer et al. used both alginate-coated chitosan and alginate-coated trimethyl chitosan NPs loaded with inactivated PR8 influenza virus for nasal immunization in BALB/c mice (Mosafer et al., 2019). Alginate is used to enhance the immunostimulatory properties and stability of chitosan and trimethyl chitosan. Although involving alginate in the coating of PR8-loaded chitosan was associated with a lesser immune response, as measured by the decreased antibody titers, it showed a significantly higher immune response when it was used for the coating of PR8-loaded trimethyl chitosan, as well as compared to the free PR8 virus (Mosafer et al., 2019). Since preserving original characteristics, such as stability and antigenic properties, is a crucial challenge in developing a nano-carrier system for proteins, alginate-nanomaterials are well suited for this task because they can be fabricated without the use of harsh conditions, such as shear force and freeze-drying, which are often used for other nano-carriers (Sarei et al., 2013).

Moreover, researchers have incorporated other types of loaded and free NPs with therapeutic potentials (e.g., metallic NPs) onto alginate-based hydrogels and nanofibers to enhance the wound healing process and reduce the toxicity of the incorporated NPs (Dodero et al., 2021). For example, Mohandas et al. incorporated zinc oxide (ZnO) NPs into alginate hydrogels to produce bandages for wound dressings. The presence of ZnO NPs on the composite bandages decreased their swelling ratio, controlled the degradation profile, and enhanced the blood clotting ability (Mohandas et al., 2015). Due to the synergistic action of alginate and ZnO NPs, an antimicrobial effect for the composite bandages was reported against *Staphylococcus aureus*, methicillin-resistant *S. aureus* (MRSA), *Escherichia coli*, and *Candida albicans*. At lower concentrations of ZnO NPs, tested *in-vitro*, these bandages were not toxic to human dermal fibroblast cells, while they induced keratinocyte infiltration toward the wound area during re-epithelialization in an *ex-vivo* porcine skin model (Mohandas et al., 2015). In another similar example, Shalumon et al. also used ZnO NPs but with sodium alginate and polyvinyl alcohol (PVA) to prepare composite nanofibers by electrospinning (Shalumon et al., 2011). The alginate-PVA fibers with lower ZnO NPs concentrations were less toxic. The alginate/PVA/ZnO NPs mats exhibited good cell adhesion when studied *in-vitro* with L929 cells, and their antibacterial activity against *S. aureus* and *E. coli* correlated with the presence of ZnO NPs (Shalumon et al., 2011).

A study by Ibrahim et al. (2020) reported on a method for the production of alginate-based tamoxifen/silver NPs with dual core-folate decorated shell for *in vitro* breast cancer therapy. The loading of silver NPs and tamoxifen within the alginate core allowed for the co-delivery of both components, altering the mechanism of action of tamoxifen. Also, due to the water solubility and pronounced surface charge of alginate, it efficiently prevented silver NP aggregation. This polymeric nanocomposite allowed for passive targeting and resulted in higher accumulation in tumors and lower toxicity to healthy tissues. In these hybrids, folic acid was used as a ligand for intracellular active targeting, which facilitated the active uptake of NPs by the tumor cells. Finally, the superior cytotoxic effect of this nanocomposite against breast cancer was concluded to have proceeded *via* ROS-driven NF-κB pathway modulation (Ibrahim et al., 2020). The incorporation of metallic NPs into alginate has also opened the door for alginate to be involved in developing specific biosensors. An amperometric glucose biosensor was developed by Buk et al. using alginate as the immobilization matrix (through covalent crosslinking) for glucose oxidase and copper oxide NPs (Buk et al., 2017). The constructed biosensor had a high sensitivity, which could be attributed to the ability of alginate to create an efficient chemical/physical membrane for the interfering molecules present in serum samples (e.g., ascorbic acid and uric acid). Also, alginate can increase the adhesiveness of the electrode and biocompatibility of the microenvironment for the immobilized glucose oxidase. Indeed, alginate influenced the development of a friendly environment for the enzyme, which resulted in satisfactory reproducibility of this biosensor (Buk et al., 2017).

Due to their relatively inexpensiveness and good removal efficiency of dyes, alginate nanocomposites have received increasing attention in recent years. Since natural alginates have a significant number of functional groups on the surface, they can easily adsorb both the anionic and the cationic dyes from wastewaters, as well as from both acidic and basic media (Heybet et al., 2021). On the other hand, adding other nanomaterials, such as GO or carbon nanotubes, is one of the most effective methods to improve the adsorption capacity of alginate-based nanomaterials (Zhuang et al., 2016; Khan et al., 2021). For example, a study by Zhuang et al. (2016) presented the fabrication of GO/alginate double and single network nanocomposite beads (GAD and GAS, respectively). GAD exhibited a higher thermal stability and specific surface than GAS, and also a greater capacity to remove methylene blue from water (Zhuang et al., 2016). In another study, a novel iron oxide/activated charcoal/β-cyclodextrin/alginate nanocomposite was fabricated also for the elimination of methylene blue from water (Yadav et al., 2020). The results indicated that the nanocomposites exhibited pronounced adsorption capacities for the elimination of cationic dyes. Approximately 99.5% of methylene blue was efficiently removed by nanocomposites within 90 min. Spherical morphologies, hydrophobicity, and cross-linked surface structure of the nanocomposites have enabled their easy recovery without any significant weight loss (Yadav et al., 2020). Similarly, a study by Nasrullah et al. (2018) has demonstrated the synthesis of activated carbon-alginate beads composite, which was applied also as a highly efficient adsorbent for removing methylene blue from aqueous solutions. These findings revealed that the obtained composite had a high surface area and therefore a high adsorption capacity for the removal of cationic dyes (Nasrullah et al., 2018).

In addition to the above mentioned applications for alginate-based nanomaterials and their potential properties, alginate can be employed for the removal of heavy metals that cause poisoning, and it can be an effective co-adjuvant in remedies for food poisoning (Pereira and Cotas, 2020; Omer, 2021). Therefore, alginate-based nanomaterials are commonly used with other natural polymers and products. The success of alginate-based nanomaterials has also been demonstrated through the possibility to fabricate hybrid nanocomposites of alginate polymers with other natural polymers (e.g., chitosan and hyaluronic acid) and with bioactive substances, especially those with low bioavailability (e.g., curcumin) (Li et al., 2008; De Santis et al., 2014; Zhang and Zhang, 2021). The next sections include an extensive up-to-date review of all the available literature that conveys the efforts of researchers in developing alginate-based nanomaterials loaded with various bee products or their extracts, as well as the relevant studies about alginate and bee products.

### Honey-Loaded Alginate-Based Nanomaterials

#### Honey and Its Medicinal Properties

Honey is produced by bees. This sweet substance is collected from the floral nectar, the secretions of aphid family members as they feed on plant sap (e.g., honeydew), or the secretions of parts of the living plant (White, 1978; Pita-Calvo and Vázquez, 2018). Bees consume flower pollen as a protein source and nectar as an energy source as much as they can to support their metabolic activities during foraging (Paray et al., 2021). The vast majority of the nectar gathered, on the other hand, is headed for regurgitation, digestion, and storage as honey and beebread (Wright et al., 2018). The substance is also to be stored as a long-term food supply for adult and larval bees to consume, e.g., during cold weather or food shortage (Carroll et al., 2017). Honey is naturally “produced” by bees, predominantly through beekeeping of *Apis mellifera* and Meliponinae. Each kind of beekeeping has its distinct characteristics and is domesticated for honey production and agricultural pollination purposes. *Apis mellifera* (*A. mellifera*) of the genus Apis, generally known as the honeybee or western/European honeybee, is one of the most prevalent honey bee species (Moritz et al., 2005). Humans introduced the species in the early 1600s, and it quickly expanded over North America, South America, Australia, New Zealand, and eastern Asia (Carpenter and Harpur, 2021). Although it is thought to have originated in Asia or Africa, it can be found in practically every continent, as it organically spread from Africa through the Middle East and Europe (Han et al., 2012). Humans have actively cultivated this species as a source of sugar. Additionally, they are invaluable pollinators of some crops, such as berries, cherries, and almost, which are exclusively dependent on pollination (Hung et al., 2018). Their natural migration with good adaptability features, as by coordinating colony cycles with the time of available local floral resources, creating a tight cluster during winter and exhibiting an improved foraging activity in arid locations, allow them to survive under any circumstances, which is favorable for domestication and large-scale production (Parker et al., 2010). At least 33 subspecies of honeybees have outbred each other. Nonetheless, the population has been declining in recent years due to multiple factors, including parasitic mites, diseases, and herbicides. Stingless bee, on the other hand, is one of the most prominent bees with at least 500 species (Rasmussen and Cameron, 2010). They can easily find a wide variety of tropical or subtropical locations across the world, including Africa, Southeast Asia, Australia, Central, and South America. Long before the introduction of honeybee, the stingless bee species were the primary source of honey in the tropical regions. Worldwide, there are more colonies of the latter than of the former (Vit et al., 2013). However, it had been abandoned due to its limited honey output and is nowadays exclusively found in tropical countries. Nowadays, the growing interest in honey for its medicinal values has brought stingless bees back into the spotlight. They have been kept on a vast scale, such that they are actively farmed by humans (Michener, 2013). Aside from that, pollinators like them play a critical role in preserving the local flora and dispersing tropical plant seeds worldwide (Grüter, 2020). The honey produced by stingless bees contains less sugar and more water than the honey produced by honeybee (Rao et al., 2016)*.* Stingless bees store their honey in wax and resin pots, while honeybee use wax structures called honeycombs (Rao et al., 2016). In addition, stingless bees exhibit several unique traits, including the preference for low camp flowers and the tendency to form tiny flocks (Vit et al., 2013). Unlike honeybee, stingless bees are also simpler to handle, less likely to be selective in forming a colony hive, and less vulnerable to illness. As such, they are accessible to industrial farming to increase the honey production (Abd Jalil et al., 2017). Nevertheless, both types of bees and their products (e.g., honey, pollen, and propolis) have been regarded as the source of revenue for generations.

A large number of studies on the chemical and biological properties of honey have been performed due to their potent antibacterial, antioxidant, anti-inflammatory and wound healing effects. Since honey has a long-standing tradition with good medicinal value, “folklore” beekeepers and honey enthusiasts have been attached to the putative medicinal effects of honey (Hadagali and Chua, 2014). However, its therapeutic effects depend on its quality, which is attributed to many factors, including the maturation of the bee nest or hive during the harvesting season. In general, the quality of honey is measured by the various chemical, physical, microbiological and sensorial characteristics (Zulkhairi Amin et al., 2018). It also depends on the location of collection, where the local environment and the floral abundance may affect the climate change. These changes have evolved suitable adaptations of the bees to their respective environments. These factors might be the reason for the diverse color, flavor, and functional properties of honey (Bänsch et al., 2020). Honey produced by honeybees and stingless bees consists in unique and distinct biological and therapeutic properties. Further details about the physicochemical and biological properties of honey produced by honeybees and stingless bees are shown in an interesting comparative review by Rao and others (Rao et al., 2016).

DeMera and Angert (2004) reported that honey from *Tetragonisca angustula* (stingless bee) and *A. mellifera* showed a significant antimicrobial activity towards *C. albicans* and *Saccharomyces cerevisiae* and intermediate susceptibility to *P. aeroginosa* and *Bacillus cereus*. In another study, *T. angustula* was documented to possess good antimicrobial activity against *S. aureu*s (Miorin et al., 2003). As for antidiabetic properties, honey from western bees showed a higher percentage of inhibition against α-amylase and α-glucosidase enzymes (Krishnasree and Ukkuru, 2017). Stingless bee honey from *Geniotrigona thoracica* was proved to prevent the increase in the levels of fasting-blood glucose, total cholesterols, triglycerides, and low-density lipoprotein. Besides, it increased the high-density lipoprotein and serum insulin levels (Aziz et al., 2017). Honey from *A. mellifera* possesses anticancer properties, as it increased the number of viable human hepatoma (HepG2) cells in a model of the hepatocellular carcinoma treatment and reduced both the rate of its growth and the tumor size. At the same time, it improved the total antioxidant status (Hassan et al., 2012). Honeybee produced honey with a bacterial flora in the conjunctival sacs of patients with cataract, who were scheduled for vitrectomy, before it was successfully eradicated after 7 days (Cernak et al., 2012). Meanwhile, stingless bee honey reduced the infection time for the eye diseases caused by *S. aureus* and *P. aeruginosa* (Ilechie et al., 2012). Honey from *Apis dorsata* (honeybee) increased intromission and ejaculation percentage in rats and increased the rate of fertility and mating (Mohamed et al., 2013). In another work, it increased testicular, epididymal weights and sperm count, quality, motility, and viability in nondiabetics (Budin et al., 2017). Mosavat et al. (2014) mentioned that this honey reduced cortisol and increased the progesterone levels in stress-induced female rats.

Skin, the largest human organ, is able to heal itself, but healing can be difficult in the presence of some injuries and diseases. In general, delayed acute and chronic wounds manifest impaired healing and often fail to convert to normal stages of healing. Due to a postponed, uncoordinated and incomplete healing process, the wounds usually descend into the state of pathologic inflammation (Guo and DiPietro, 2010). Chronic wounds are related to diabetes mellitus, ischemia, venous stasis disease or pressure (Zhao et al., 2016). Herein, honey is found to be the best natural wound healer. Honey has been targeted to clear bacteria, malodor, and debridement (Hixon et al., 2019). Ancient Romans, Assyrian, Egyptians, Greeks and Chinese utilized honey as a topical treatment for wounds and skin illness. A study by Al-Waili (2001) reported that a topical application of 90% diluted crude honey showed a significant improvement of symptoms in seborrheic dermatitis patients. In another study, honey mixture treatment in combination with corticosteroids remarkably reduced the symptoms of psoriatic and atopic dermatitis patients (Al-Waili, 2003). Silver sulfadiazine (SSD) is used to treat burn wounds by absorbing wound exudates and releasing the antimicrobial silver into the wound site (Khansa et al., 2019). Jull et al. (2015) reported that honey dressings healed partial thickness burns more rapid as compared to conventional dressings and to those treated with SSD. They found that honey heals a mixed population of acute and chronic wounds more rapidly than SSD or sugar dressings. The anti-inflammatory properties possessed by honey have prompted a remarkable improvement in wound healing. In the same report, honey was also found to heal rapidly infected post-operative wounds, pressure ulcers, and Fournier’s gangrene.

#### Honey and Alginate

Honey is the most common bee products used with alginate-based nano- and non-nanomaterials for various pharmaceutical and biomedical applications, especially in wound dressing and other tissue engineering and regenerative medicine approaches (Table 1).

**TABLE 1:** List of studies that included honey in alginate formulations.

Study	Final product	Fabrication method	Other polymers/compounds	Application/therapeutic effects of the final product (or main findings)
Tang et al. (2019)	Nanofibrous membrane	Electrospinning	Polyvinyl alcohol (synthetic polymers to increase the electrospinnability and the mechanical strength of alginate)	Bioactive, antioxidant, antibacterial and non-toxic wound dressing (*in-vitro*)
Datta et al. (2018)	3D scaffolds	Complexation followed by extrusion 3D printing	—	Bioinks with improved cell responses for future applications as bioprinted tissue engineered constructs (*in-situ*)
Mirzaei et al. (2018)	Hydrogel	Ionic crosslinking	—	An ointment accelerates wound healing (rat model) with potent antibacterial and antibiofilm activities
Nazeri et al. (2015)	Hydrogel	—	—	Wound healing (rat model)
Barui et al. (2013)	Fibrous matrix	Wet spinning	—	Enhanced re-epithelialization in keratinocytes (*in-vitro*); increased the reduction in wound gap with improved cellular viability, enhanced promoted expressions of Ki67, p63 and its isoforms (TAp63, ΔNp63) as well as E-cadherin
van der Weyden, (2005)	Hydrogel sheet	—	—	Antibacterial, anti-inflammatory and deodorizing dressing for venous ulcer healing (case study)
Mukhopadhyay et al. (2020)	3D hydrogels	Dual cross-linking (ionically and covalently)	—	Antimicrobial and wound healing (*in-vitro* and mouse model)
Saberian et al. (2021)	Hydrogel	Interpolyelectrolyte complexation	Chitosan (a biopolymer with good gelling properties for wound healing) and *Aloe vera* extract (a natural ingredient effective in wound healing)	Recommended for future applications in wound healing and skin tissue engineering
Barui et al. (2014)	Fibrous matrix	Ionic cross-linking	—	Enhanced cell viability and expressions of cell-cell adhesion molecule (E-cadherin) and prime molecules of extracellular matrix (Collagen I and III) by HaCaT and 3T3 (*in-vitro*)
Azam and Amin, (2018)	Hydrogel films	Ionic cross-linking	—	The water vapor transmission rates of honey-alginate hydrogels are equivalent to that of commercial wound dressings like OpSite and Metalline
Ligouri and Peters, (2009)	Dressings	—	—	Wound healing (case study)
Webb, (2009)	Dressings	—	—	Wound healing (case study)
Milne, (2008)	Dressings	—	—	Wound healing (case study)
Baldos et al. (2021)	Hydrogel dressings	Ionic cross-linking	—	Antioxidant and antibacterial (*in-vitro*)
Datta et al. (2021)	3D scaffolds	Ionic cross-linking followed by extrusion 3D printing	—	Enhanced fibroblast 3T3 proliferation and adhesion (*in-vitro*)
Lee et al. (2021)	Dressings	—	—	Wound healing
Asa et al. (2013)	Hydrogel dressings	Ionic cross-linking	—	Recommended for wound healing based on their physicochemical properties

In a recent study of nanofibers, honey/alginate/PVA nanofibrous membranes were synthesized using the electrospinning technique and examined for their potential as a bioactive wound dressing material (Tang et al., 2019). The acacia honey used in this study was harvested in Shaanxi province, China. Since it is difficult to electrospin pure alginate due to its high electrical conductivity, PVA was added to improve electrospinnability and the mechanical strength of alginate. The results of the size analysis, as performed using the scanning electron microscopy (SEM), revealed that adding honey to the nanofibers increased the diameter of the fibers and the spinnability of the mixture. The lower electrical conductivity and viscosity of honey/alginate/PVA solutions could be due to the corresponding reduction of the alginate content. The nanofibers without honey (alginate/PVA) had a smooth morphology and homogeneous dimension, while with 20% (v/v) honey content, they had a less uniform morphology with a broader distribution of nanofiber diameters. Fourier-transform infrared spectroscopy (FTIR) analysis also showed that honey was successfully incorporated in the nanofibers. Since water absorption capability is an essential property for wound recovery, the increment of the honey content in nanofibers led to the water absorption ratio dropping accordingly. Based on the weight loss results, this may result from the high water-solubility of honey. Similarly, the increase in the honey content caused the increment of weight loss of the membrane, suggesting that the honey content of the nanofibers determined the ability of exudate management. The assessment of the nanofibrous membranes with an increased concentration of honey confirmed their potential in wound healing with improved antioxidant and antibacterial properties. The honey possessed an outstanding radical scavenging activity due to the abundance of phenolic antioxidants, as showed by the radical scavenging activity using DPPH (2,2-Diphenyl-1-picrylhydrazyl) solutions and quantitated using an ultraviolet-visible spectrophotometer. The antibacterial activity of the membranes was studied using the disc diffusion and dynamic contact assay. The results showed that the inhibition zones against *E. coli* and *S. aureus* in disc diffusion assay became increasingly clearer and larger with an increased honey content in the nanofibers. The same trend was observed in the dynamic contact assay of honey/alginate/PVA. Additionally, honey/alginate/PVA nanofibrous membranes have good biocompatibility (Tang et al., 2019). Thus far, the nanofibrous membranes with honey have the potential for use as effective wound dressings.

Although they did not produce nanoscale materials, other studies have shown the successful incorporation of honey in alginate hydrogels and the therapeutic potentials of the resulted composite. Mirzaei et al. developed and analyzed alginate-based honey hydrogel to investigate its potential as an ointment in rat burn wound healing (Mirzaei et al., 2018). The honey exposed to gamma-rays for sterilization was secured from three provinces in Iran, Damavand, Semnan and Ardebill. The hydrogel consisting in alginate-based honey was formulated using CaCl_2_ as the ionic crosslinking agent. Even at 75% concentration in normal saline, honey was linked to alginate using CaCl_2_. The hydrogel was prepared from alginate salts in honey with suitable concentrations of bacteria including *S. aureus, Klebsiella pneumoniae, Acinetobacter baumannii* and *P. aeruginosa*. Since the antibacterial effect for Damavand honey was greater than that for others, it was used for alginate-based hydrogel preparation. The results demonstrated a potent antibacterial activity of the hydrogel, which may be attributed to the type of bees’ feeding (e.g., Thyme plant). The floral nectar possessed a thymol active ingredient, which enhanced the antibacterial activity of Damavand honey. The findings on the healing activity of the hydrogel in infected wounds demonstrated a promising effect compared to the control group with pure honey. Indeed, the hydrogel-honey was shown to accelerate the healing process in 14 days. All the infected wounds were healed at the end of the 14-day interval following the hydrogel use. The alginate-based honey hydrogel was also revealed to have better healing effects against biofilm-forming bacteria on burned wounds than those of the hydrogel alone (Mirzaei et al., 2018). Herein, the combination of alginate and honey hydrogel seems a promising natural product for burn wounds that are resistant to antibiotics, protecting the individuals from the bacterial growth.

In another study, Baldos and group investigated the effect of various radiation treatments on the honey-alginate wound dressing (HAWD) (Baldos et al., 2021). The honey utilized in this study was collected from the beehives of stingless bees, *Tetragonula biroi*, locally known as “lukot” in Sta. Maria, Laguna, Philippines. The honey used for the production of HAWD also came from the same origin. The preparation of HAWD involved sodium alginate with 2% CaCl_2_ solution. The finished product was irradiated with various doses of radiation for sterilization. The effects of radiation on HAWD were studied using several physicochemical parameters of honey, such as total soluble solids (TSS) and total phenolic and flavonoid components. The results demonstrated that the quantity of TSS in HAWD was not substantially altered at any dosages of irradiation, which was in line with the typical moisture content of stingless bee honey. There was no significant change in the pH or the flavonoid content among the irradiated samples. Unlike the flavonoid levels, the phenolic content rose dramatically with irradiation dosages, perhaps owing to radiolytic destruction of hydrolyzable tannins in honey. The study also indicated that irradiating honey had minimal physicochemical effects, but no antibacterial effectiveness against *S. aureus* at the majority of radiation dosages. Nonetheless, HAWD sterilized with an electron beam at 25 kGy proved adequate for eradication of the microbiological contamination. The authors concluded that the future usage of irradiated HAWD as a natural product-based replacement for commercial wound care treatments should be studied (Baldos et al., 2021). The effects of radiation-sterilized honey alginate wound dressing on the exudating wound were done by Asa et al. by assessing its physicochemical properties (Asa et al., 2013). Since calcium-alginate wound dressings have the capacity to gel, the authors believed that the cross-linked alginate gels might absorb wound fluid while also keeping the wound region wet. A honey alginate wound dressing was designed and irradiated for sterility after being combined with the antimicrobial characteristics of honey and the absorption and gelling capabilities of alginate. The results from the study revealed that the substance had a lower pH (4.40 ± 0.02), which is better for wound healing, than alginate dressings alone (5.40 ± 0.04). In addition, the former had a low moisture content (10.25 ± 1.11%). Meanwhile, the water vapor transmission rate (WVTR) of the sterilized honey alginate dressings showed a general rise in 48 h, but a rapid absorption rate when applied on the wound fluid. The influence of irradiation on the tensile strength of the material was negligible. The radiation-sterilized honey alginate wound dressing physicochemical properties, such as acidic pH, absorbency, moisture vapor permeability, and absorption rate, established its suitability as a wound dressing for exuding wounds. Because the product had a low moisture level, it is expected to have a longer shelf life (Asa et al., 2013).

The effect of honey-based alginate hydrogel, alginate hydrogel and commercial alginate dressings in wound recovery in a rat model was also assessed (Nazeri et al., 2015). However, the details of the location where the utilized honey was procured, the method of formulation and the characteristics of the honey-loaded alginate hydrogel were not addressed. A total of 20 male Wistar rats were divided into four groups of five. After the excision of the wound, the animals were exposed to the treatments until 21 days. One of the rats in each group was euthanized on the fourth, seventh, 14th, and 21st day and skin samples were taken for histopathological analysis (i.e., Hematoxylin-Eosin staining (H&E) and Masson’s trichrome staining). The outcomes showed that the average total time of wound healing in the group treated with honey-based alginate hydrogel dressing was the least as compared to the other groups. The time required for wound healing in honey-based alginate hydrogel, positive control, free alginate hydrogel, and negative control groups was 7, 8.1, 8.3, and 10.2 days, respectively (*p* < 0.05). The wound healing effects of the honey-based hydrogel as compared to other samples were attributed to the synergistic effect of the hydrogel and the honey components. According to the authors, honey-based alginate hydrogel is considerably more practical as a wound dressing for the treatment of superficial wounds (Nazeri et al., 2015). This seems as a potential alternative for wound healing dressing, certainly paving the way for further study and development.

Towards a further understanding of the cellular and molecular mechanisms underlying the wound healing effects by honey-based alginate hydrogels, several *in-vitro* studies were conducted using different models. Barui et al. examined the molecular events involving the keratinocyte population during re-epithelialization when exposed to the honey-alginate matrix using an *in-vitro* 2D epidermal wound-healing model (Barui et al., 2013). The honey used in this study was collected from the beekeepers of greater Kolkata. The honey-alginate matrix was formulated through the scaffold fabrication technique. The commercialized sodium-alginate was mixed thoroughly with raw honey (multiflora origin) in a 1:1 ratio and the honey-alginate fiber was formed by wet spinning in a CaCl_2_ bath where CaCl_2_ ions were used as the crosslinking agent for alginate. However, details on the characteristics of the honey-alginate fibers were not mentioned. The wound healing assessment was performed on human epidermal keratinocyte (HaCaT) cells with exposure to the sterilized honey-alginate fiber matrix and alginate fiber. The live/dead cell was examined to analyze the wound closure status. Using real-time PCR and immunocytochemistry techniques, the primary expression of Ki67, p63, and E-cadherin, along with the percentage of change in cellular electrical impedance, were examined. The authors reported that compared to alginate alone, the honey-alginate fiber matrix showed a faster reduction of the wound gap and ameliorated gene expressions involved in cellular viability, proliferation and cell-cell adhesion. The results indicated that honey-alginate fiber matrix was comparatively better than alginate fiber in facilitating re-epithelialization of the wounded HaCaT population. Overall, the effective honey-combined alginate fiber matrix significantly improved the wound healing progression as compared to the free alginate fiber (Barui et al., 2013).

Mukhopadhyay et al. fabricated commercialized honey from India in sodium alginate hydrogels to investigate its potential in wound healing, as compared to non-honey blended sodium alginate hydrogels (Mukhopadhyay et al., 2020). Alginate hydrogels were fabricated using 10 wt% aqueous stock solution of sodium-alginate with 2, 4, 6 and 10% honey through a dual cross-linking method (ionically and covalently). The authors asserted that the honey might have provided some value to the intermediate stiffness of the fabric and its regular swelling properties, which may also prevent the polymer from degrading erratically and provide a favorable environment for the proliferation of cells. The surface morphology of honey-sodium alginate and free sodium alginate hydrogels characterized using SEM changed with the increase in honey concentration. The addition of honey to the hydrogels resulted in an atypical globular microstructure that crystallized at a given concentration. While sodium alginate hydrogels exhibited a nearly flat surface shape, 4% honey-sodium alginate hydrogel showed the ideal microstructure for cellular adhesion. The optimum honey concentration (4%) showed sudden steepness in swelling behaviour and reached an equilibrium state; this concentration is required to replace huge amount of intramolecular free water of hydrogel. Additionally, the crystalline structure and mechanical properties of honey-sodium alginate hydrogels, especially the 4% one, showed that the cross-linked intramolecular arrangement tends to be more organized than that in the sodium alginate hydrogels. Indeed, the honey enzymes amylase and glucosidase weakened the amide bond established during covalent crosslinking, resulting in a reduction in the stiffness of the hydrogel. The gel property also remained constant because the calcium-alginate ionic crosslinking was not disrupted. All soft hydrogels for topical application on skin wounds demonstrated a considerable hysteresis, but no reversible deformation. Moreover, honey-sodium alginate hydrogel demonstrated an increase of the swelling index and pore dilation in correlation with an increase in the honey concentration, which can be explained by the fact that honey, being hygroscopic in nature, increased the water uptake capacity of the hydrogels, leading to the formation of an aqueous environment conducive to degradation, thereby alleviating cell growth, proliferation, and adhesion. Interestingly, all honey-sodium alginate hydrogel samples tended to reach an equilibrium within 10 days except the 4% honey-sodium alginate hydrogel, which degraded in a controlled manner with a particular steepness and without reaching any stable state. The overall findings demonstrated adequate extracellular matrix deposition, negligible fibroblast migration to the granulation tissue after matrix remodeling, unwounded skin resembling epithelialization, and minimal scar length, with the 3D hydrogel substrate containing 4% honey producing the optimal healing environment. The 4% honey-sodium alginate hydrogel also had a decent cellular viability (HaCaT and 3T3) and antimicrobial potential against MRSA and *E. coli*, highlighting its medicinal value. Notably, following the invasive (histopathology) and non-invasive (Swept Source Optical Coherence Tomography) imaging of wound contraction kinetics *in-vivo*, it was evident that 4% honey-sodium alginate hydrogel treated wound closure in the murine model attained the epithelial thickness similar to unwounded skin (Mukhopadhyay et al., 2020). In conclusion, this study showed that the structurally modified dual cross-linked alginate hydrogel embedded with honey could be an excellent agent for tissue engineering and antimicrobial wound healing.

Another study by Barui et al. also investigated the biocompatibility of honey-alginate and alginate fibrous scaffolds in HaCaT and 3T3 (mouse embryonic fibroblasts) cells with primary molecular expressions (Barui et al., 2014). Raw honey of multi-floral origin was obtained and physically embedded in cross-linked alginate networks. In order to maintain the integrity of the fibrous scaffold-based mix and spinnability, a 1:1 volume ratio of alginate to honey was applied in the procedure. The authors asserted that the properties of honey during the fabrication process were preserved, which could be due to the cross-linking of the fiber in the physiological environment. It was also concluded that the mechanical weakness and poor cell adhesion of alginate could be improved when combined with natural polymers, such as honey. In this study, it was evident from the SEM analysis that honey prevents the honey-alginate fiber shrinkage, while the surface texture of alginate fibers showed a crack-like structure upon drying. Additionally, a mechanical test on the tensile strength of honey-alginate fibers came up with four times lower values than those on alginate fibers, indicating that the honey-alginate fibers had a lesser stiffness, which is favorable for cell attachment. Further corroborating the observation, the methylthialazole tetrazolium (MTT) assay showed a higher cell viability in up to 72 h culture on the honey-alginate scaffold as compared to alginate alone. The same trend was observed upon assessing the cell-cell adhesion molecule (E-cadherin) and the prime molecules of extracellular matrix (Collagen I and III) in HaCaT and 3T3. In conclusion, the overall results indicated that the honey-alginate fibrous matrix is a superior choice for tissue engineering applications, warranting further investigation (Barui et al., 2014).

Datta et al. investigated 3D-printed alginate/honey scaffolds that could be implemented in *in-situ* skin tissue engineering (Datta et al., 2018). A scaffold using 0.5% commercial honey (from Dabur, India) and 5% alginate was compared to a scaffold containing only 5% alginate. The physiochemical characteristics of the bioink, such as pH, conductivity, and viscosity, changed significantly, notably in honey/alginate. The viscosity analysis of both samples was within the acceptable printing range. However, honey-embedded alginate had a lower viscosity than pure alginate, which might be attributed to the components in honey being incorporated into the alginate polymer chains, producing chain tangling and lowering the mixture viscosity. The pH and conductivity of honey-containing solutions were similarly reduced by incorporating honey in the alginate solution. The research also revealed that combining honey at low concentrations with alginate bioinks improves the cell responsiveness and irregular breakdown of the scaffolds, while retaining the physicochemical features that might benefit soft tissue engineering applications and wound healing management (Datta et al., 2018).

Moreover, a honey-alginate dressing was also used for the treatment of a venous leg ulcer. A case study by van der Weyden documented a 6-month treatment of chronic venous ulcers with a honey-based alginate dressing as the alternative to the current wound management therapies (van der Weyden, 2005). This indicates that the honey-based alginate dressing is a valuable alternative to traditional therapies and has long-lasting effects. The honey alginate dressing used was a commercialized product from New Zealand plant *Leptospermum scoparium* (Manuka). The product applied to the patient was in the form of a sheet that could be cut to the size and shape of the wound. The patient experienced no discomfort or pain after 10 days of the treatment using the honey alginate dressings, while the necrotic area entirely debrided. The dressings frequently changed, every 3–4 days, to avoid the exudate from the wound around the damaged wound area. The wound size decreased over time and completely healed by 28 weeks. This study indicated that honey-alginate composite effectively acts as the antibacterial, anti-inflammatory and deodorizing dressing, with total healing of the ulcer achieved (van der Weyden, 2005).

Milne reported a case study aimed to examine the changes in wound size and the presence of nonviable tissue following a treatment with honey-impregnated calcium alginate dressings (HICADs) and non-impregnated calcium alginate dressings (NICADs) (Milne, 2008). A 42-year-old man reported several lower extremity ulcerations that started as bullous lesions and progressed to severe deep ulcers with violaceous, undermined, and irregular margins and erythematous halos. Histopathology of the biopsied lesions revealed an ulcerated suppurative chronic inflammatory disease with granulation tissue, leading to a provisional diagnosis of bullous variant pyoderma gangrenosum. HICADs were used on the medial calf wounds, whereas NICADs were used on the lateral calf wounds. Dressings were wrapped with non-adhesive foam, fastened with gauze, changed every other day, or as needed for strike-through drainage. According to the findings of this investigation, autolytic debridement seemed to be comparable between NICADs and HICADs. Compared to NICADs, all HICAD wounds showed a faster decrease in the wound area. In conclusion, HICADs may hasten wound healing due to their antibacterial properties and other therapeutic qualities not seen in NICADs (Milne, 2008).

Two studies presented at the 41st Annual Wound, Ostomy and Continence Nurses Conference investigated the effects of *Leptospermum* honey alginate dressing as a novel product on various types of wounds, including the MRSA colonized wound. In the first study, wound measurements, pain reports, wound bed state, and exudate levels were recorded to assess the product’s efficacy (Ligouri and Peters, 2009). The authors opted to employ this product in their wound program because of the evidence and the difficulty of mending chronic non-healing wounds despite the abundance of wound solutions currently accessible. The trial included four chronic wound patients. These wounds had a surgical, pressure, venous, or traumatic origin, which matched their treatment population. Atraumatic dressing changes with minimal pain were observed in all patients with improved wound size and bed status, as well as management of exudate. This study concluded that the use of *Leptospermum* honey improved wound healing and patient and clinician satisfaction. The authors suggested that active *Leptospermum* honey calcium alginate dressings might assist a broad range of inpatient and outpatient wound managements (Ligouri and Peters, 2009). The second study briefly reported the effects of *Leptospermum* honey-impregnated calcium alginate dressings on a mentally compromised patient with MRSA colonization of a dehisced abdominal wound (Webb, 2009). The patient was recruited from the San Mateo Medical Center, with a non-healing MRSA infested dehisced surgical lesion. Patient education regarding local wound care using *Leptospermum* honey–impregnated calcium alginate dressings was a part of preparing the patient for mental health discharge. The patient was instructed to clean the wound daily and apply a *Leptospermum* honey calcium alginate dressing and a cover. This psychiatric patient’s dressing change was made simple by using a *Leptospermum* honey dressing over a dehisced abdominal location. The patient could easily follow the treatment, and the previously non-healing wound began to heal. Conclusively, calcium alginate dressings with *Leptospermum* honey are practical, cost-efficient infection control and can be the alternative for wound management in the clinic (Webb, 2009).

On the other hand, in order to enhance the properties of honey-alginate, researchers incorporated honey into alginate hydrogels with other potential biopolymers or natural products with known therapeutic effects. Considering their potential positive effects, Saberian et al. fabricated a hydrogel made of alginate and chitosan combined with honey and *Aloe vera* (*Aloe barbadensis* Miller) for the application as a wound dressing (Saberian et al., 2021). The honey used was obtained from the mountainous area of Layezangan, Fars, Iran, whereas *Aloe vera* was gathered from the suburbs of Tehran, Iran. No details on bees were mentioned in this report. The alginate-chitosan hydrogel was produced through the modified inter-polyelectrolyte complex method. The properties of honey-embedded hydrogels were conducted by comparing to other groups with additional substances and the control (i.e., a hydrogel with honey or *Aloe vera*, and a free hydrogel). The combined honey and *Aloe vera* embedded hydrogels exhibited a surface with porous-like cavities which were almost identical in shape and size to the regular networks, suggesting that the distinctive structural properties of hydrogels were preserved. Most of the cavities had an interconnected structure, making them suitable for cell growth and migration and re-epithelialization and cell proliferation in a wound closing process. Although alginate-chitosan-*Aloe vera*-honey hydrogel had a poorer structure than alginate-chitosan-*Aloe vera* hydrogel, it possessed all the necessary properties to facilitate wound healing. The WVTR performance of hydrogels in this study was considerably lower than the optimal range of WVTR for wound healing (∼2000 g/m^2^/24 h). This may be a drawback of this study since variations in the concentrations of components destroyed the hydrogel structure. Nevertheless, given the other essential features of hydrogels, the authors assumed that these hydrogels were effective when used for *in-vivo* investigation. The results of the WVTR also revealed that all groups exhibited hydrophilic qualities based on the contact angle measurements. The outcomes from the antibacterial activity test showed a substantial and suboptimal degree of inhibition against *S. aureus* and *S. aeruginosa*, respectively, as measured on the alginate-chitosan-*Aloe vera*-honey combination. The level of inhibition against *S. aureus* was precisely identical to gentamicin. Results of the MTT assay and the viability percentage also indicated that hydrogels with honey and *Aloe vera* were the most biocompatible and could cause the least cytotoxicity. Also, the natural components (honey and *Aloe vera*) employed in the hydrogel decreased the RBC hemolysis to a safe level. As it was inferable from the SEM images, the hydrogels combined with honey and *Aloe vera* showed a sustainable cell adhesion, which was corroborated by the cell-ECM interactions and their role in fibroblast adherence to the hydrogel surface, being a necessary step for the healing process to commence. This study illustrated that a combination of honey and *Aloe vera* with an alginate/chitosan hydrogel maintains structural aspects in cellular interactions of wound healing dressings (Saberian et al., 2021).

Lee and co-authors compared the wound healing effects of honey-containing alginate (Medihoney alginate, Derma Sciences, United States) to standard alginate dressing (Algisite M, Smith & Nephew, United Kingdom) in chronic wounds (Lee et al., 2021). The experiment was conducted on patients with chronic lower extremity wounds despite more than 3 months of wound care, from January 2019 to January 2021. They were treated with either Medihoney alginate (*n* = 21) or Algisite M (*n* = 16). The initial wound was assessed, and microorganisms from the wound were cultured. The antibiotics were administered, and their duration depended on the wound condition. The results revealed that the rate of wound surface area reduction on patients treated with honey-containing alginate dressing was significantly greater than for those treated with an alginate-only dressing, indicating that honey had a beneficial effect when combined with alginate for the treatment of chronic wounds of the lower extremities. Indeed, honey-containing dressings are effective in wound healing because they have antibiotic, antioxidant, and anti-inflammatory effects, which are desirable factors to promote wound healing. This study further supports the notion that a significant number of wounds colonized with antibiotic-resistant bacteria could be treatable with the medical-grade honey containing alginate (Lee et al., 2021).

In a study aimed to fabricate and characterize sodium alginate-hydrogel films incorporating Manuka honey (Azam and Amin, 2018). Honeybees produce this honey from the nectar that they collect from New Zealand mānuka tree flowers. Manuka honey is a well-studied natural product that has exhibited various medicinal properties of interest. In 2007, the U.S. Food and Drug Administration (FDA) approved Manuka honey as a recommended alternative remedy from a natural origin for wound healing. Among the unique features of Manuka honey, there is a high level of methylglyoxal (MGO), which is an effective antibacterial agent (Johnston et al., 2018). In Azam’s study, the Manuka honey was procured from Nature’s Way, New Zealand. Both the WVTR and swelling ratio of the hydrogel films were measured, while the gel fraction was investigated for the mechanical properties. The results revealed that adding Manuka honey to the alginate film exhibited reduced swelling. Hence, the swelling percentage of the alginate hydrogel film was at the maximum at 512 ± 21% and decreased to 197 ± 9% when alginate hydrogel film contained 10% (v/v) Manuka honey. The honey-loaded alginate hydrogel film had the lowest gel fraction (12 ± 1%), as compared to the alginate hydrogel film, which had a gel fraction of 45 ± 3%. The alginate hydrogel films containing Manuka honey exhibited an increase in Young’s modulus at low Manuka honey concentrations. However, tensile strength, tensile strain, and Young’s Modulus were low for the alginate hydrogel films with greater Manuka honey concentrations. The results also revealed that the WVTRs of all honey-alginate hydrogels were equivalent to those of commercial wound dressings, such as OpSite or Metalline. In short, alginate hydrogel films containing honey exhibit desirable qualities and may be used in the management of wound healing (Azam and Amin, 2018).

The leading UK-based company “Advancis Medical,” which is an advanced wound care specialist manufacturer, had early realized the promising potential of incorporating medicinal honey in alginate wound dressings by producing a novel product, Algivon^®^ (Algivon^®^). Algivon^®^ is composed of alginate fibers impregnated with 100% medical-grade Manuka honey. The alginate fibers enable a sustained and slow-release of honey, which inhibits the clinically relevant pathogens and biofilms found in wounds and stimulates cells involved in wound repair, including fibroblasts and keratinocytes (Bulman et al., 2017). Therefore, Algivon^®^ is considered a natural antimicrobial with no known resistance. It is a safe and effective debridement and desloughing agent, and an ideal choice for wetter wounds, given that alginate itself has a small capacity to absorb water, meaning that the honey is not washed away with the exudate. Also, the dressing is very soft and conformable, ideal for cavities and for debriding and desloughing large areas of necrotic and sloughy tissue.

A novel honey-embedded bioink was fabricated using alginate and commercial honey from India (Dabur, No. BD0793) by 3D bioprinting (Datta et al., 2018). Adding honey to the alginate bioink altered both the physiological and biological properties of the pure alginate bioinks. The study was done to evaluate the printability of honey-alginate bioinks. The bioink formulations displayed the viscosity of honey-alginate solutions, which decreased as the concentration of honey (1–5% w/w) increased. The viscosity reduction could be due to further penetration of honey components in the alginate chains, which significantly reduces polymer chain entanglements. In order to obtain mechanically robust constructs, the viscosity range was maintained at a higher alginate concentration (5% w/v). Similarly, the increase of the strut size corresponded to an increased honey concentration, but the opposite applied to the pore area. The addition of honey to the bioinks also revealed defined bioprinted shapes, but only in compositions with less than 5% of honey. For a robust evaluation of print fidelity, the circularity bioinks were analyzed, suggesting low printability of bioinks with a high concentration of honey. The results confirmed that 1 and 2% of honey formulations in honey-alginate are considered the most acceptable bioprinted scaffolds. These novel bioinks seem to be applicable for skin tissue engineering in clinical settings (Datta et al., 2018).

From another point of view, honey crystallization is a natural phenomenon. Because consumers prefer liquefied honey, beekeepers often have to liquefy this product before making it accessible on the market. Drawing from that situation, using free and immobilized enzyme invertase, Souad and others presented a simple approach to keep honey liquid while enhancing the nutritional content (Souad et al., 2018). The honey was harvested in Algeria’s Wilaya of Souk Ahras. Invertase, the enzyme utilized in this investigation, was isolated from baker’s yeast (*Saccharomyces cerevisiae*). The enzyme invertase was immobilized *via* the encapsulation approach in calcium alginate gel. To examine the quality of honey in free and immobilized states, the total polyphenol and flavonoid contents, and its antioxidant activity were recorded and analyzed. The honey samples containing crystals were separated into three groups: honey only, honey-free enzyme, and honey-immobilized enzyme. The size of alginate beads directly impacted the effectiveness of enzymatic catalysis, with fine and homogeneous diameters that may be acceptable for the industrial scale. This study showed that a 2 mm bead had an average immobilisation rate in the excess of 98.5%, which fulfilled some critical parameters for invertase immobilization. The study also revealed that the total polyphenol content in honey with the free enzyme was higher than that of honey alone, but comparable to honey with the immobilized enzyme. Similarly, the flavonoid content in the former was considerably higher than that of honey alone, but comparable to a sample with the immobilized enzyme. Furthermore, the moderate antioxidant activity measured in both honey-containing samples was lower than that measured in the untreated honey. Indeed, the treated honey was shown to undergo considerable compositional alterations, notably in its total content of polyphenols and flavonoids. In sum, honey treated with an enzyme, particularly when immobilized in a calcium alginate gel, seems to be a promising technique to improve the quality of honey in markets (Souad et al., 2018).

### Propolis-Loaded Alginate-Based Nanomaterials

Propolis, often known as bee glue, is a brownish-green resinous material accumulated by bees from different buds, leaves, crusts, and other plant components. The properties of propolis are most often influenced by its botanical origin and the bee species producing it (Zhang et al., 2019). Propolis is high in essential oils and phenolic components, as well as antibacterial, antioxidants, anti-tumoral action, anti-inflammatory compounds. It has a wide range of uses in food preservation and is beneficial to human health (Keskin et al., 2019). Despite the fact that propolis contains numerous bioactive compounds (e.g., phenolic acids, flavonoids, ketones, and aldehydes), its use as a food additive is limited due to its low oral bioavailability; studies have shown that orally administered propolis is rapidly degraded in the body, particularly in the gastrointestinal tract, making it a rather good candidate for use as a therapeutic substance in the biomedical field and wound treatment. The four phases of the wound healing process include haemostasis, inflammation, proliferation (granulation-contraction), and remodelling. Propolis is involved in mediating the first stages of wound healing through several mechanisms, such as haemostasis and inflammation. It promotes the expression of transforming growth factor (TGF), a protein that controls practically all cellular processes (Aranci et al., 2020; Dalponte Dallabona et al., 2020). Two mechanisms may be delineated in terms of its antibacterial action. The first works directly on the microbe, while the second stimulates the immune system, resulting in the activation of the organism’s inherent defenses, allowing it to combat infection. Furthermore, according to various studies, propolis has no toxicity or negative effects in animal models or people, allowing it to be employed in biomedical applications such as wound healing scaffolds (Zhang et al., 2019). Furthermore, the strong taste and scent make direct application difficult; hence, new techniques of harnessing the propolis potentials are being pursued. Due to the presence of a wide range of bioactive chemicals, it has a lot of potential in the food sector, too. Propolis is also used in the cosmetics, food, and agricultural industries (Hegazi et al., 2021). Therefore, several studies have used alginate-based nano- and non-nanomaterials to enhance the bioaccessibility and bioavailability of propolis. These studies have highlighted a great potential benefit of propolis combined with alginate for the human health and deserve further research for potential pharmaceutical applications (Table 2).

**TABLE 2:** List of studies that included propolis in alginate formulations.

Study	Final product	Fabrication method	Other polymers/compounds	Application/therapeutic effects of the final product (or main findings)
Zhang et al. (2019)	Nanoparticles	Complexation	Zein and caseinate proteins	Propolis enhanced the bioaccessibility nanoparticles and their stability towards aggregation
Xuan et al. (2020)	Nanoparticles	Oil-in-water emulsion	Whey protein isolate and α-tocopherol	Improved α-tocopherol stability
				Recommended for multi-nutraceutical delivery
Hegazi et al. (2021)	Nanoparticles	Ionic cross-linking	—	Improved the immunity (increased the immunoglobulin titers and reduced the pro-inflammatory cytokines) of newborn goats
Hegazi et al. (2019)	Nanoparticles	Ionic cross-linking	—	Antimicrobial (*in-vitro*)
Sadek et al. (2020)	Nanoparticles	Ionic cross-linking	—	Improved the health status of newborn goats
Keskin et al. (2019)	Microcapsules	Ionic cross-linking	—	The release profile of microcapsules indicate that they can increase the bioavailability of bioactive components of propolis
Dalponte Dallabona et al. (2020)	Beads	Ionic cross-linking	Jabuticaba fruit peel	Antioxidant
				Suggested as an oral delivery route or as a natural colorant in food or drinks
Aranci et al. (2020)	3D scaffolds	Ionic cross-linking	—	Antibacterial and wound healing (*in-vitro*)
Cruz et al. (2021)	Solution	Bilayer coating	—	Antioxidant and antibacterial
Homem et al. (2021)	Films	Ionic cross-linking	Gelatin	Antibacterial
Asri et al. (2020)	Fibrous scaffolds	Electrospinning	Cellulose	Recommended for skin tissue engineering applications based on their cytotoxicity structural porosity and permeability
Cesar et al. (2021)	Dressings	Emulsion cross-linking	All-trans retinoic acid (vitamin A) and Tween 80 (surfactant)	Promoting anti-inflammatory action in an erythrocyte membrane stabilization model

A study by Zhang et al. presented a simple one-step procedure to fabricate propolis-loaded zein/caseinate/alginate NPs (Zhang et al., 2019). A well-blended solution containing deprotonated propolis, soluble zein, dissociated sodium caseinate micelles (NaCas), and alginate was prepared at an alkaline pH, after which this alkaline solution had 0.1 M citrate buffer (pH 3.8) added to it to produce composite NPs. Propolis was provided by Bee Words Industry Co. Ltd in Zhejiang Province, China. These NPs were shown to be aggregation-resistant throughout a broad pH range (2–8) and salt concentration range (0–300 mM NaCl). The bioaccessibility of propolis encapsulated with NPs was raised to 80% as compared to free propolis. The findings revealed a viable clean and scalable technique for encapsulating hydrophobic nutraceuticals for food, supplement, and pharmaceutical applications (Zhang et al., 2019). In another study, whey protein isolate encapsulated into Brazilian propolis-alginate complex particles were synthesized at pH 7.0 and characterized for size distribution, zeta-potential, antioxidant activity, and propolis encapsulation (Xuan et al., 2020). The results showed that propolis and/or alginate increased tocopherol stability during storage, but sodium alginate counteracted the beneficial impact of propolis on the vitamin stability during enzymatic hydrolysis. The information gained might lead to further research on using biopolymer-stabilized emulsions for multi-nutrient storage and delivery (Xuan et al., 2020).

A study by Keskin et al. used sodium alginate to encapsulate the ethanol extract of propolis (Keskin et al., 2019). Raw propolis samples were collected in the Turkish towns of Artvin and Zonguldak. Ten grams of raw propolis were then combined with 100 ml 100% ethanol and shaken for 24 h at a regulated pace. The combination was then filtered using a filter paper and vacuum. The propolis extract was made from the clear filtrate obtained. The release characteristics of propolis-loaded alginate beads were tested in simulated gastric and intestinal systems. The researchers reported that the bioactive components of propolis might reach the upper section of the large intestine, according to the release characteristics of the beads. This scenario may also boost the bioavailability of certain nutrients. The authors came to the conclusion that beads might be used as a dietary supplement or as a food additive in the food sector (Keskin et al., 2019). Turkish propolis was also used in producing wound dressings based on the 3D printing technology (Aranci et al., 2020). The propolis extract was supplied by Scientific Bio Solutions, Istanbul, Turkey. Propolis-integrated sodium alginate scaffolds in various combinations and architectures were produced. The antimicrobial activity against numerous harmful bacteria (*E. coli* and *S. aureus*) was also tested, and it was shown to be outstanding. The scaffold was further subjected to an *in-vitro* cytotoxicity test utilizing the extract technique on the human dermal fibroblasts (HFFF2) cell line, demonstrating its nontoxic nature. It was concluded that the propolis-alginate scaffolds, which were 3D printed, could be useful structures for wound dressing applications owing to their unique features (Aranci et al., 2020). These findings were consistent with a study aimed to synthesize polymeric films out of biodegradable and biocompatible polymers as delivery vehicles to increase the stability and long-term efficiency of a propolis extract from Portugal in wound healing (Homem et al., 2021). A simple, green solvent casting/phase inversion approach was used to make propolis extract-containing sodium alginate/gelatin films, which were then cross-linked with CaCl_2_ solutions. The antibacterial activity was also tested. The findings validated the modified films’ capacity to combat bacterial infections produced by *P. aeruginosa* and *S. aureus*, as well as their ability to treat infected wounds. Because of their superior bacterial suppression, the findings implied that propolis extract-loaded alginate/gelatin films might be utilized as an alternative to standard therapies for infected wounds (Homem et al., 2021).

A recent study by Cesar et al. evaluated the physical characteristics and release kinetics of a PVA and sodium alginate blend foam for wound dressing loaded with propolis and *all*-trans retinoic acid (Cesar et al., 2021). Green propolis was purchased in the form of a lyophilized powder from Apis Flora® (Ribeirao Preto, SP, Brazil). SEM scans revealed a porous structure with a variety of morphologies. The FTIR spectra revealed a strongly cross-linked structure, with hydrogen bonds and acetal bridges connecting the two polymers. Physical properties of the wound dressings obtained were sufficient. The findings showed that the anti-inflammatory properties were unaffected by the manufacturing method. The rate of release was quick enough to enhance biological processes such as the anti-inflammatory activity. However, the impact was minor compared to typical medications (Cesar et al., 2021). A group of researchers from Iran prepared an electrolytic solution of carboxymethylcellulose, calcium alginate, and New Zealand Manuka propolis, then used it to generate adequate fiber diameters with uniform size distribution (Asri et al., 2020). To determine the scaffold characteristics, the researchers used SEM, FTIR, and the mean of weight reduction. For assessing the cellular viability of the samples, an MTT test and a mechanical test were conducted on the specimens. The rate of disintegration was linear, and the scaffolds were completely destroyed after 21 days. They concluded that calcium carboxymethylcellulose/calcium alginate/propolis scaffold had a desirable structure for porosity and permeability, making it an excellent candidate for use in skin tissue engineering (Asri et al., 2020).

In the last 3 years, three research articles from Egypt showed promising findings from studying the medicinal properties of alginate NPs loaded with propolis collected from an apiary farm near El-Mansoura City, Egypt. The first research by Hegazi et al. aimed to assess the antibacterial efficacy of propolis-encapsulated alginate NPs against several harmful bacteria (Hegazi et al., 2019). Propolis was successfully encapsulated inside alginate NPs with good characteristics; this was confirmed by the transmission electron microscopy (TEM), FTIR, zeta potential, and dielectric spectroscopy. The obtained propolis-alginate NPs were spherical, discrete and had a small particle size (13 nm). Compared to pure propolis and/or antibiotic (clindamycin), the propolis-alginate NPs demonstrated the greatest antimicrobial activity against all pathogens tested. They are said to have displayed a synergistic antibacterial activity against multiple bacterial strains (Hegazi et al., 2019). The second study by Sadek et al. evaluated the possibility of using propolis-alginate NPs as a natural additive or as a replacement for colostrum to improve the health status of newborn Egyptian Nubian goat and avoid the increasing rate of mortality under the natural system (Sadek et al., 2020). The controlled gelation approach was used to fabricate the propolis-alginate NPs and TEM was used to analyze their morphological characteristics. The newborn goats were given colostrum by their mothers or artificially fed colostrum, as well as crude propolis or propolis-alginate NPs. The goats were weighed twice a week and their daily body weight increases were kept track of. The body weight, milk intake, and milk chemistry of the goats were all measured. It was found out that nano-encapsulation of propolis inside alginate NPs enhanced the health of goats even at low doses. Furthermore, this material was suggested as a substitute for colostrum in order to enhance the performance of newborn goats (Sadek et al., 2020). Thereafter, the latest study of this research group was also on newborn Egyptian-Nubian goats, specifically on the potential role of propolis-alginate NPs in boosting and strengthening their immune systems (Hegazi et al., 2021). Due to its excellent functional characteristics, propolis was chosen as a natural colostrum ingredient, while alginate nanoparticles were used as a novel medication carrier for the oral administration of propolis. Similar to their previous study, the propolis-alginate NPs were fabricated and characterized, and the high-performance liquid chromatography (HPLC) was used to determine the flavonoid content of propolis. Like in the previous study, the newborn goats were given colostrum by their mothers or artificially fed colostrum, as well as propolis or propolis-alginate NPs, while the goats were weighed twice a week. After the treatment with propolis and propolis-alginate NPs, serum immunoglobulins G and A (IgG and IgA), serum total protein, and serum cytokine levels were measured at various treatment times. The study showed that pure propolis had a weaker impact on IgG and IgA levels and cytokine levels than propolis-alginate NPs. The findings indicated that propolis-alginate NPs improved the health of the goats, boosted immunoglobulin titters, and lowered pro-inflammatory cytokines (Hegazi et al., 2021). Therefore, it might be concluded from these studies that propolis-alginate NPs can be employed as health and immune boosters for newborns. Establishing an effective propolis oral delivery nano-system on an industrial scale with the use of alginate NPs is possible.

Professor Estacio supervised a group of undergraduate students at the College of Veterinary Medicine, University of the Philippines Los Baños to study the healing activity of different propolis-alginate wound dressings in mice and cats as final year projects. Propolis produced from Philippine stingless bees (*T. biroi Friese*) was used to fabricate propolis-alginate dressing. This dressing was then used for excisional wound healing in mice and it showed a significantly accelerated healing processes. Histopathologic observation of healed wounds indicated complete re-epithelialization with hair follicles and sebaceous glands formation, normal dermal thickness, mild epidermal thickening, and the absence of scarring after using propolis-alginate dressing (Rada, 2016). In another project, they used the same dressing on sutured wounds of domestic shorthaired cats and it showed a similar healing activity and histopathological findings. The authors reported good healing without any discernible sutured wound outline at the shortest duration of healing, with minimal scabbing (Resma, 2016). In addition, they indicated that propolis-alginate dressings were the most cost-effective treatments of choice in both projects (Rada, 2016; Resma, 2016). In their third project, they used both gamma radiated-honey and propolis of the same bee species in fabricating two formulations of alginate fibers for a mouse model of incisional wound (Virtucio, 2014). However, the full results of this project are not available.

Moreover, propolis-loaded alginate-nanomaterials could play a potential role as an excellent natural preservative of the native properties of nutrients and foods. Dallabona et al. have encapsulated anthocyanins and phenolic compounds extracted from propolis from Tubuna (*Scaptotrigona bipunctata*) stingless bees and a native Brazilian fruit peel-jabuticaba (Plinia cauliflora (Mart.) Kausel) in an alginate hydrogel (Dalponte Dallabona et al., 2020). Propolis was obtained from a native Atlantic Forest in Maringá (south of Brazil) and extracted with a number of steps. Ionotropic gelation was used to form the alginate hydrogel, which was done by dripping it into a CaCl_2_ solution. The authors show that anthocyanins and the extracted phenolic compounds could be encapsulated into the alginate hydrogel beads with high efficiency, implying that they might be used as an oral delivery route or as a natural colourant additive in food or drinks (Dalponte Dallabona et al., 2020). In another study, the impact of a sodium alginate bilayer coating integrating the green propolis extract on the shelf life, physical and chemical characteristics, microbiological parameters, and sensory acceptability of *Colossoma macropomum* fillets was evaluated (Cruz et al., 2021). Raw green propolis was collected in apiaries in Betim, Minas Gerais, Brazil, and utilized to make hydroalcoholic extracts. The antioxidant and antibacterial properties of green propolis extracts were found to be promising. Gas chromatography mass spectrometry (GC–MS) was used to identify 27 metabolites, the majority of which were terpenoids. The primary ingredient of the green propolis extract was cyclolaudenol, which was discovered for the first time in green propolis extracts. The sodium alginate bilayer coating on *C. macropomum* fillets resulted in great sensory acceptability, decreased microbiological degradation, and a longer shelf-life (up to 11 days) under cold storage. Taken together, these findings suggest that green propolis extracts might be an excellent natural preservative for fish coating (Cruz et al., 2021).

### Alginate-Based Nanomaterials Loaded with Other Bee Products

A few studies have also shown the potential of alginate-based nano- and non-nanomaterials in loading less widely known bee products (Table 3).

**TABLE 3:** List of studies that included other bee products other than honey or propolis in alginate formulations.

Study	Bee product	Final product	Fabrication method	Other polymers/compounds	Application/therapeutic effects of the final product (or main findings)
Pakolpakcil and Draczynski, (2021)	Bee Pollen	Nanofibrous mat	Electrospinning	Polyvinyl alcohol	High biocompatibility
					Recommended for various biomedical applications such as wound dressing, tissue engineering, and drug delivery systems
Lee et al. (2018)	Bee Venom	Nanoparticles	Oil-in-water emulsion	Span 80 and Tween 80 (surfactants)	An adjuvant and delivery vehicle for PRRSV vaccine. Induced non-specific immune stimulating actions, particularly those related to Th1 responses and viral clearance activities against PRRSV infection (pig model)
Smuga-Kogut et al. (2020)	Beebread caviar	Capsules	Ionic cross-linking	—	Classified as probiotic food
Xing et al. (2003)	Bee Venom	Gel beads	Coating	Liposomes	A high-capacity drug carrier (*in-vivo* and *in-vitro*)
PRRSV, porcine reproductive and respiratory syndrome virus					

One bee product other than honey or propolis that has the potential to affect human health is bee pollen. It is used based on its biological and therapeutic properties that include antioxidant, anti-carcinogenic, antibacterial, anti-inflammatory, and anti-allergic activities (Denisow and Denisow-Pietrzyk, 2016). Bee pollen is produced by stingless bees and honey bees collecting floral pollen from flowers and seedlings, as well as nectar or honey, bee secretions, wax, and enzymes. It comprises proteins, carbohydrates, lipids and amino acids under biologically acceptable conditions (Anderson et al., 2014; Mohammad et al., 2021). There has not been any research on the development of bee pollen-loaded alginate-based nanomaterials or any type of polymeric nanofibers until Pakolpakçıl and Draczynski (in 2021) have hypothesised that combining bee pollen with alginate to create green nanofibrous materials might be a viable bioengineering option (Pakolpakçıl and Draczynski, 2021). They used an electrospinning process to create Polish bee pollen-loaded alginate-based nanofibrous mats and studied their shape, chemical content and thermal characteristics. SEM, FTIR, and differential scanning calorimetry (DSC) were used to examine the green electrospun nanofibrous mats. The SEM images of the electrospun bee pollen-loaded alginate-based mats showed primarily nanoscale features, with a very few flaws. The FTIR spectrum showed that bee pollen had been incorporated into the sodium alginate and PVA nanofiber mats. Adding bee pollen to the alginate-PVA polymer solution generated a considerable shift in the glass transition temperature, but had no influence on the melting temperature, according to the DSC thermogram. The authors indicated that the approach for producing bee pollen-loaded alginate-based nanofibrous material using green electrospinning of water-based systems might be an alternative. They showed how ecologically friendly nanofibrous materials might be created for a variety of uses, including biomedical applications, such as wound dressings and scaffoldings (Pakolpakçıl and Draczynski, 2021).

Furthermore, the mixture of bee pollen, bee salivary enzymes, and regurgitated honey fermented by indigenous microbes is another bee product, which is colloquially called beebread (Mohammad et al., 2021). Smuga-Kogut et al. invented a unique buckwheat honey beebread caviar (collected in Stonio Apiary, West Pomerania, Poland) through immobilization with sodium alginate (Smuga-Kogut et al., 2020). The beebread originated from the Jeyce apiary, comprising 180 bee colonies near a buckwheat crop (Darlowo, West Pomerania, Poland). This novel food product was created by cross-linking buckwheat honey with a 2% solution of sodium alginate (ratio 1:1) as the carrier, using the immobilization process commonly known as spherification in molecular gastronomy. It is a systematic approach to making food items more visually appealing while maintaining their nutritional qualities. The resulting caviar was in the form of little silky gel balls with a liquid inside any flavor. The beebread immobilized in this manner showed a longer supply of nutrients, bioactive elements, and a superior flavor. Furthermore, this beebread may be ingested regularly to correct vitamin deficits and avoid numerous diseases. Because this product is manufactured using an immobilization approach with alginate, it may be kept for an extended period of time while retaining the beneficial features of the beebread. The nutritional benefit of caviar balls for ingestion was confirmed by microscopic examination. The immobilization of the beebread encased by alginate reduces the preparation time. Additionally, storing the product for a longer duration of time increases the antioxidant characteristics of the caviar balls. It was shown that increasing the storage duration improves the phenolic content of the caviar. The study of the beebread caviar also revealed that it contained 0.34 mg GAE/ml extract and was stable for 5 days after manufacture. After another 5 days, the phenolic compounds had decreased in concentration, but remained twice as high relative to the unprocessed product. The caviar had a total acidity of 33.7 mval/kg and a 22.53% extract content. It also had a high overall sensory score of 4.8 out of a possible 5 points. Because beebread includes a high lactic acid concentration, beebread caviar may also be categorized as a probiotic food. This novel, appealing and easy method of beebread ingestion in the shape of the caviar might become one of the products of pleasant and valuable cuisine (Smuga-Kogut et al., 2020).

Moreover, royal jelly is one of the most promising bee products, which has a great nutritional and therapeutic value and has been used as a food and a medicine. The hypopharyngeal glands of young worker bees create royal jelly, often known as a superfood, which is only supplied to the queen bee for the remainder of her life (Kim et al., 2010; Park et al., 2011). Freshly collected royal jelly has a yellowish to white colour and is high in proteins, free amino acids, lipids, vitamins, and carbohydrates (Pasupuleti et al., 2017). Royal jelly contains around 185 chemical components, according to contemporary spectrometric research (Shaiqah et al., 2020), while the most significant protein found in royal jelly is royalactin (Pasupuleti et al., 2017).

Owing to its unique properties, royal jelly has the potential to heal a variety of human diseases. Its antioxidant, anticancer, antiaging, neurotropic, anti-inflammatory and wound-healing properties are proven (Siavash et al., 2015). It has been shown that under the influence of royal jelly, human fibroblasts were able to migrate and raise sphingolipid levels while lowering collagen production and creation in both *in-vivo* and *in-vitro* wound-healing models (Kim et al., 2010). As a result, royal jelly decreased the time it took for desquamated skin lesions to heal (Kim et al., 2010). Another research on the usage of royal jelly found that it protects human skin against photoaging caused by ultraviolet B rays by boosting collagen formation (Park et al., 2011). Aside from traditional therapies, royal jelly dressing is an efficient technique to treat diabetic foot ulcers. This is owing to its vasodilation actions in the wound area, which may assist in widening the blood vessels and improving the blood flow. Because of its antibacterial properties, it also aids in the prevention of infections (Siavash et al., 2015). To the best of our knowledge, there has been no study on loading royal jelly in any alginate-based nanomaterials. However, a study by Shaiqah et al. intended to microencapsulate royal jelly in alginate beads and use a quality-by-design (QbD) technique to enhance royal jelly beads formulation (Shaiqah et al., 2020). They utilized royal jelly from Kuala Lumpur, Malaysia’s Giant B Honey. They used a 2^4^ factorial design with three center points (19 runs) to optimize alginate-royal jelly microbeads electrospray parameters, including high voltage, flow rate, tip-to-collector distance, and nozzle size, while the responses were particle size distribution, particle size, and sphericity factor. The reactions of each run were analyzed using Design-Expert^®^ software. They discovered that the gelation technique of extruding the solution by electrospraying into a CaCl_2_ solution results in the creation of royal jelly in alginate beads. Design of experiments (DoE) was used to test the electrospray operating parameters. The size of the nozzle had a considerable impact on the particle size, but not the particle size distribution, as the sphericity factor was heavily influenced by the flow rate. As a result, this research demonstrated that various parameters influence distinct royal jelly properties in alginate beads (Shaiqah et al., 2020).

Bee venom is a good example of physiologically active peptide medicines with a lot of therapeutic promises, but it has a lot of drawbacks too, such as a short plasma half-life after intravenous infusion and the inability to identify the precise dosages following traditional bee sting treatments (Xing et al., 2003). Bee venom is a bitter, colourless liquid with a variety of proteins that promote local inflammation and function as anticoagulants in the active fraction. Bee venom is a complex combination of enzymes and low-molecular-weight polypeptides, and it has been found to include a variety of enzymes (Borumand, 2013). Both fresh and dried bee venom mostly vary in terms of volatile components; nonetheless, the biological activities are comparable overall. They include carbohydrates, enzymes, amino acids, and minerals as well as anti-inflammatory and inflammatory chemicals (Lee et al., 2018). Bee venom has been utilized to treat a variety of cardiac ailments. It has been used to treat patients with a number of degenerative nervous system ailments, including multiple sclerosis and Parkinson’s disease. In cell and animal studies, bee venom was also shown to be effective against a variety of malignancies, with a higher cytotoxic activity against cancer cells as compared to most normal cells (Saeed et al., 2013; Wehbe et al., 2019; Duffy et al., 2020).

To overcome the drawbacks of bee venom and enhance its biological and therapeutic potentials, researchers proposed using biopolymers loaded with bee venom. Only two studies used alginate-based nanomaterials. A study from Korea aimed to construct a novel encapsulated bee venom (*Apis mellifera*) material using alginate and chitosan NPs that have mucoadhesive properties and can gently release the bee venom to effectively stimulate the systemic immune response (Lee et al., 2018). The authors tested whether the newly created chitosan/alginate NPs encapsulating bee venom with slow-release characteristics and mucosal adhesiveness might boost the systemic immune response and increase the clearance of swine reproductive and respiratory syndrome virus in pigs through the nasal route. To assess the robust immune-stimulating effects of the two distinct NP-encapsulated bee venoms, they vaccinated the individuals with a modified live porcine reproductive and respiratory syndrome virus (PRRSV) vaccine after administration. Both groups showed a considerable rise in T-helper cell numbers, according to the researchers. They came to the conclusion that nasal application of chitosan/alginate-bee venom might provide good protection against the PPRS infection (Lee et al., 2018). The second study was from China, where the researchers investigated a novel formulation for oral administration using coated calcium alginate gel beads-entrapped liposome and bee venom peptide (Xing et al., 2003). They employed gamma scintigraphy to demonstrate the transit of the dosage form through the gut and trace the times at which it left the stomach and arrived at the colon. Gamma scintigraphy may be used to capture a timelapse photographic series of pictures when used to test oral medication administration. The scientists discovered that the quantity of bee venom released from the coated calcium alginate gel beads-entrapped liposome was affected by the calcium and sodium alginate concentrations, as well as by the amount of bee venom in the liposome and in the coating. The arrival period of the pills in the colon was shown to be 4–5 h. They concluded that a coated calcium alginate gel beads-entrapped liposome is a viable technology for colon-specific medication delivery.

Bee venom contains more than 15 different peptides and proteins with the most interesting of them being the cytolytic peptides melittin and phospholipase-A2 (Ağan and Kekeçoğlu, 2020). Melittin is a basic 26-amino-acid polypeptide, and its chemical formula is C_131_H_229_N_39_O_32_ (Chen et al., 2016). The accumulated data indicate that melittin is a potential candidate for cancer therapy, as it causes suppression of tumor hallmarks, including anti-apoptosis, cell cycle progression, migration, metastasis, inflammation and angiogenesis. Studies have indicated that it modulates the hallmarks of cancer through multiple signaling pathways such as the Janus kinase 2/signal transducer and activator of transcription 3 (JAK2/STAT3), NF-κB, hypoxia-inducible factor 1 subunit alpha (HIF-1ɑ), and vascular endothelial growth factor (VEGF) (Jo et al., 2012; Liu et al., 2016; Mansour et al., 2021). Therefore, Rady et al. (2017) suggested that the use of nanotechnology-mediated delivery of melittin could enhance the therapeutic efficacy of melittin in addition to providing a more effective route for the systemic delivery to target cancer cells with minimal or none hemolytic effect. In one of the studies reporting on the development of nanocarriers for melittin, researchers from Taiwan and Japan have successfully synthesized ionically cross-linked (by CaCl_2_) oligopeptide-alginate NPs (Wattanakul et al., 2019). They used commercially available melittin from Sigma-Aldrich, United States, and they compared it with DOX in terms of loading efficiency and cytotoxicity. It was shown that the oligopeptide side chain in alginate induces a hydrogen bonding interaction with peptide groups in the melittin structure, but the alginate backbone does not cause such a specific interaction with melittin. Therefore, the loaded amounts of melittin and DOX were more than 200 and 10% higher in oligopeptide-alginate NPs than in free alginate NPs, respectively. This difference was attributed to the interaction between the DOX-active amine group and the carboxylic acid group of alginate. The results of the cell viability assay revealed that 80% of Caco-2 cells (human colorectal adenocarcinoma) did not survive under the 2.5 μM dose of melittin-loaded oligopeptide-alginate NPs, while free melittin caused almost no damage to Caco-2 cells at the same molar concentration. However, only 0.4 μM of DOX caused 50% cell death, and the oligopeptide-alginate NPs did not enhance the performance of DOX (Wattanakul et al., 2019). The involvement of alginate-based nanomaterials in preparing bee venom delivery systems has a great promise since it has been successfully used with other natural cytotoxic agents and venoms. For example, in a study by Saeed et al., sodium alginate NPs were prepared and encapsulated with ICD-85 as a cytotoxic agent. Their *in-vitro* cytotoxicity was analyzed on the proliferation of the HEp-2 cell line (Saeed et al., 2013). The ICD-85 is a combination of three peptides, ranging from 10,000 to 30,000 Da, derived from the venoms of snake (*Agkistrodon halys*) and scorpion (*Hemiscorpius lepturus*) and it was provided from Razi Vaccine and Serum Research Institute, Karaj, Iran. The researchers used an ionic gelation approach to synthesize the ICD-85 loaded alginate NPs, which were then evaluated for particle size, zeta potential, TEM, FTIR, and *in-vitro* release. The MTT assay was used to assess *in-vitro* cytotoxicity. ICD-85 loaded NPs had spherical forms with a size of around 200 nm, according to TEM. Their zeta potential was calculated to be –16.1 mV. The loading capacity and encapsulation efficiency, respectively, were 90.48 and 90.24%. The *in-vitro* release profile showed persistent release patterns, with an early burst of activity followed by a gradual release (Saeed et al., 2013).

On the other hand, researchers from the University of Delhi, India performed a research on Indian spectacle cobra (*Naja naja*) venom and Russell’s viper (*Daboia russelii*) venom (Bhattacharya et al., 2014). Venoms were lyophilized and kept at 4°C in an amber-colored vial until needed. Relevant venoms were weighed, diluted in 0.9% saline, and utilised at suitable dilutions for the tests. The researchers entrapped various components of the antivenom in alginate as a cheap and biodegradable polymer option, so that the functional properties of the antivenom were maintained even after the intestinal absorption, exhibiting venom neutralizing-effects both *in-vivo* and *in-vitro*. Their research might lead to the creation of an effective first-aid treatment for snake envenomation, enhancing the victim’s chances of survival (Bhattacharya et al., 2014). Although the abovementioned studies by Saeed et al. and Bhattacharya et al. have not involved bee venom, they could still seize researchers’ attention to alginate-based nanomaterials as potential carrier systems for bee venom or its extracts of active molecules such as enzymes or peptides.

Indeed, despite the promising potential role of alginate-based nano- and non-nanomaterials in loading bee products, there is still a long way towards considering these nanoproducts as the next generation of natural treatments for many human diseases. Although alginates have attractive physicochemical properties, they also have some disadvantages that may limit their use in fabricating carriers for bee products or their extracts. For example, studies have shown that alginates have a poor dimensional stability (cannot be stored for a long time), poor tear strength (need to be combined with other biopolymers), the ability to depolymerize upon heating at over 60°C (loss of viscosity), unpleasant odor, the ability to precipitate at low pH, incompatibility with heavy metals, ion-leaching leading to instability, limited control of mechanical properties, and less accurate reproduction of structural details as compared to elastomers, while also being relatively messy to work with and not useful for dry wounds (may need wetting before removal) (Nandini et al., 2008; David et al., 2016; Puscaselu et al., 2020).

On the other hand, it is indisputable that bee products are promising natural candidates for various applications among complementary and alternative medicines, notwithstanding that they could be contaminated with potentially toxic elements, such as heavy metals (e.g., lead, cadmium and mercury), pesticide residues (protectors of plants from weeds, pathogenic bacteria, fungi, or insects), antibiotics, and other organic pollutants. Various studies have confirmed the presence of such elements in samples of bee products, which in turn could lead to unintended negative consequences on human health. Such elements can be introduced to bee products accidentally by environmental hazards or intentionally by beekeeping practices, for example by adding antibiotics or medications to the hive to control infection in bees and prevent the development of disease (Al-Waili et al., 2012; Islam et al., 2014; El-Nahhal, 2020). Furthermore, bee products can be contaminated by pathogenic bacteria such as *Clostridium botulinum*, which produces the dangerous “botulinum toxins” under low-oxygen conditions (Nevas et al., 2002), as well as by genetically modified organisms such as rape and maize (Bogdanov, 2006). Other disadvantages of bee products can result from their original characteristics. For example, since honey is high in sugar, the excessive topical application of honey for diabetic wounds could lead to increased blood glucose levels or dehydration of tissues (Eteraf-Oskouei and Najafi, 2013). Another example is associated with the possible risks of MGO from the bee products (especially Manuka honey and propolis), which have been raised due to the capability of this compound to glycate proteins and thus delay the healing of wounds and bone injuries, particularly in diabetic patients (Majtan, 2011; Aikawa et al., 2017). Also, allergic reactions to pollen or to bee proteins can occur in rare cases (Jagdis and Sussman, 2012; Aguiar et al., 2017). However, clinical evidence to confirm these concerns has not been reported yet. Moreover, most clinical applications would require medical devices containing bee products to be sterilized using gamma irradiation prior to use, and it is uncertain to what extent such procedures would alter the composition of honey and diminish its therapeutic potency. Therefore, although they can be considered as a green system that comes with a number of positive features, the usage of bee products or their extracts loaded into alginate-based nano- and non-nanomaterials should be studied well before moving to the application stage.

## Future Directions

Selecting bee products with rich constituents of compounds with a high potential to affect the intended physiological targets is the first and crucial step towards fabricating successful alginate-based nanomaterials. Studies seem to show that stingless bee products might have a superior therapeutic potential compared to honeybee products, given that the former are rich in a number of bioactive compounds with a wide range of medicinal properties (Al-Hatamleh et al., 2020a). Also, recent studies have shown that stingless bee products could have better nutritional and medicinal properties due to their higher antioxidant contents (Fuenmayor et al., 2013; Brown et al., 2020). This could be attributed to the fact that stingless bees’ original location is tropical regions and thus their products are mostly multifloral (Al-Hatamleh et al., 2020a). For example, research in the past 2 years has shown that stingless bee honeys from Malaysia, Australia and Brazil exhibit unique physicochemical properties (Fletcher et al., 2020; Zawawi et al., 2022). Interestingly, stingless bee honey has been reported as a novel source of rare trehalulose sugar (α-d-glucosylpyranosyl-1,1-d-fructofuranose), a structural isomer of sucrose with a sweet taste and very similar physical and organoleptic properties to sucrose (Ravaud et al., 2007). The main advantage of trehalulose over sucrose is the slower rate of release of monosaccharides into the bloodstream. Thus, it is highly beneficial because of having low insulinemic and glycemic indices, as adding up to its anticariogenic and antioxidant activities (Fletcher et al., 2020). Therefore, since the excess consumption of added sugar is associated with a variety of diseases, stingless bee honey could be one of the best natural sweeteners amid the growing need for sugar substitutes. Further, alginate-based nanomaterials could play a vital role in this regard as safe and well-studied nano-carriers, especially for oral administration. Furthermore, alginate-based nanomaterials can play a potential role as carrier systems loaded with phytochemicals extracted from stingless bee products. Among these phytochemicals, quercetin emerges as a promising compound with multifunctional properties including antioxidant, anti-inflammatory, antimicrobial, anti-carcinogenic, and psychostimulant, as well as the ability to inhibit lipid peroxidation, platelet aggregation and capillary permeability, and to stimulate mitochondrial biogenesis (Li et al., 2016; Al-Hatamleh et al., 2020a). Despite the high therapeutic potential of quercetin, it has poor solubility, bioavailability, bioaccessibility and stability, which has encouraged researchers to utilize several nano-based drug delivery systems to overcome these obstacles (Cai et al., 2013; Wang et al., 2021). Alginate-based nanomaterials as greenly synthesized nanomaterials obtainable using simple and little laborious methods are currently considered among the most ideal materials for quercetin delivery (Qi et al., 2015; Selvaraj et al., 2021). Also, alginate-based nanomaterials loaded with quercetin have revealed a desirable entrapment efficiency and release profiles, as well as superior stability, bioavailability, and biological activities (Parhi et al., 2020; Selvaraj et al., 2021).

However, the involvement of stingless bee products in fabricating various types of nanomaterials is still very limited. More specifically, almost all the bee products that were used in producing alginate-based nanomaterials had honeybee origins. Therefore, after knowing the huge potentials of alginate and stingless bee products, researchers are recommended to synthesize alginate-based nanomaterials loaded with various products of stingless bees, especially honey, and study their characteristics and biological potentials. Such studies are not only necessary to enhance the role of stingless bee products in biomedical and pharmaceutical applications, but they can also be important for producing cosmetics, supplements and foods, especially since stingless bee products are not well-known and are less used for consumption. Hence, moving these approaches from research to industry could play an important role in the innovative development of stingless beekeeping and other associated industries in tropical countries.

Moreover, the selection of an inappropriate nanoformulation method can put precious research time, materials, resources, and financial support at risk. Thus, selecting the suitable method for producing alginate-based nanomaterials loaded with stingless bee products or their extracts is of paramount importance. To overcome this challenge, it is recommended to adopt Multi-Criteria Decision Making (MCDM) models to ensure that the selections made are rooted in a scientific approach. One of the MCDM methods that can help in this regard is the Analytical Hierarchy Process (AHP). The AHP is a structured technique that decomposes a complex issue into a hierarchy constructed with a goal, criteria and alternatives, thus helping in organizing and analyzing complex decisions (Dolan, 2010). The AHP is derived from individual experts’ experiences utilized to estimate the qualitative factors through the pair-wise comparison matrix. This compensatory decision methodology is widely used for selecting suitable methods for the preparation of NPs (Velmurugan et al., 2011). The AHP has been recommended for finding out the most suitable method for the preparation of polymeric NPs loaded with specific natural product-derived compounds (e.g., camptothecin) (Krishnamoorthy and Mahalingam, 2015). Applying the AHP to fabricate alginate-based nanomaterials loaded with stingless bee products or their extracts would be a novel work and it could help in increasing the product quality and shortening the product development cycles.

Regarding commercialization prospects of nanocomposites based on alginates and bee products, it should be kept in mind that bee products are natural products and are, as such, intrinsically not patentable unless they comprise a component of a medical device alongside another compound or a material. Another scenario in which bee products may be patentable is when they are processed into a unique form, such as that of microneedles for drug delivery (Frydman et al., 2020). Therefore, the presence of alginate increases the potential for intellectual protection of the formulation and thus boosts its potential for commercialization at the clinical level. Here, of course, the more distinct and structurally complex the alginate component is, the greater the patentability of the invention and the greater the potential for commercialization. This, however, does not renounce the issue of seasonal variability in the composition of the bee products, which presents a significant barrier before the widespread use of such products in pharmacy and medicine, especially in cultures, such as the North American, that have traditionally favored the synthetic medicines over the natural ones because of the reliable composition of the former, as opposed to a greater batch-to-batch variability of the latter. Proving the compositional consistency of active ingredients presents an important factor in ensuring that bee products, including the alginate nanocomposites, are prescription-based and covered by the insurers as opposed to being only available as over-the-counter supplements. For this reason, it is conceivable that bee products will fare better commercially in a country such as Malaysia, which presents the global benchmark for successful integration of traditional and alternative medicines into the R&D network of mainstream healthcare (Muhammad and Awaisu, 2008). In future, ideally, the standard, the traditional and the alternative medicines should form a coherent and synergistic whole (Leonti and Casu, 2013). Concepts such as the nanocomposites of alginate and bee products present the essential steps toward reaching these holistic niches.

## Conclusion

This is an extensive up-to-date review of all the available literature that conveys the efforts of researchers in developing alginate-based nanomaterials loaded with various bee products or their extracts. In the light of the cited literature, this review mainly aimed to brief on alginate and its characteristics, alginate nanoformulations and fabrication methods, and the pharmaceutical and biomedical applications of alginate-based nanomaterials. Further, a special emphasis was given to studies that involved bee products in developing alginate-based nanomaterials for medicinal and other biological purposes, which, to the best of our knowledge, has not been discussed before. Although alginate-based nanomaterials have a great potential to improve the therapeutic effects of bee products as green synthesized carrier systems, studies pursuing these approaches are still limited. Also, the majority of these studies have involved either honey or propolis in the nanoformulations, and the resulted products were exclusively developed for research purposes and did not reach the industry stage yet. This review serves as a comprehensive reference for researchers who plan to conduct future research in this promising area, especially with the growing demand for using natural products (e.g., bee products) as pharmaceuticals. This, in turn, should encourage the scientific community to accelerate and support the development of green synthesized nanomaterials (e.g., alginate) to preserve the native properties of these products and to enhance their health benefits. Alginate-based nanomaterials have been proposed as potential carriers for stingless bee products and continued future research on their use in the delivery of these antioxidant-rich natural products may lead to the translation to various branches of pharmaceutical, biomedical, and food industries. Lastly, the AHP technique was suggested as a potential MCDM method to select the most suitable formulation methods for these green nanomaterials in the effort to fulfill their undoubtedly large therapeutic potentials.
